# Tracing the Evolution of Plant Glyoxalase III Enzymes for Structural and Functional Divergence

**DOI:** 10.3390/antiox10050648

**Published:** 2021-04-23

**Authors:** Brijesh Kumar, Charanpreet Kaur, Ashwani Pareek, Sudhir K. Sopory, Sneh L. Singla-Pareek

**Affiliations:** 1 Plant Stress Biology, International Centre for Genetic Engineering and Biotechnology, Aruna Asaf Ali Marg, New Delhi 110067, Indiacharanpreet06@gmail.com (C.K.); sopory@icgeb.res.in (S.K.S.); 2 Stress Physiology and Molecular Biology Laboratory, School of Life Sciences, Jawaharlal Nehru University, New Delhi 110067, India; ashwanip@mail.jnu.ac.in

**Keywords:** glyoxalase III, DJ-1_PfpI domains, evolution, horizontal gene transfer, methylglyoxal

## Abstract

Glyoxalase pathway is the primary route for metabolism of methylglyoxal (MG), a toxic ubiquitous metabolite that affects redox homeostasis. It neutralizes MG using Glyoxalase I and Glyoxalase II (GLYI and GLYII) enzymes in the presence of reduced glutathione. In addition, there also exists a shorter route for the MG detoxification in the form of Glyoxalase III (GLYIII) enzymes, which can convert MG into D-lactate in a single-step without involving glutathione. GLYIII proteins in different systems demonstrate diverse functional capacities and play a vital role in oxidative stress response. To gain insight into their evolutionary patterns, here we studied the evolution of GLYIII enzymes across prokaryotes and eukaryotes, with special emphasis on plants. GLYIII proteins are characterized by the presence of DJ-1_PfpI domains thereby, belonging to the DJ-1_PfpI protein superfamily. Our analysis delineated evolution of double DJ-1_PfpI domains in plant GLYIII. Based on sequence and structural characteristics, plant GLYIII enzymes could be categorized into three different clusters, which followed different evolutionary trajectories. Importantly, GLYIII proteins from monocots and dicots group separately in each cluster and the each of the two domains of these proteins also cluster differentially. Overall, our findings suggested that GLYIII proteins have undergone significant evolutionary changes in plants, which is likely to confer diversity and flexibility in their functions.

## 1. Introduction

Glyoxalase (GLY) pathway is well-known for its role in plant stress adaptation as well as in disease conditions in animals. The two enzymes of this pathway, glyoxalase I (GLYI) and glyoxalase II (GLYII) act by metabolizing methylglyoxal (MG), an α-ketoaldehyde produced primarily by spontaneous dephosphorylation of triose phosphates [1,2], to a relatively non-toxic compound, D-lactate. The toxicity of MG is attributed to its highly reactive nature towards proteins, lipids, and nucleic acids. Being a carbonyl electrophile, MG can readily modify arginine, lysine, and cysteine amino acids of proteins as well as adenine, cytosine, and guanine residues of nucleic acids, to form advanced glycation end products (AGEs) in turn, altering the functional properties of these biomolecules [3]. Besides triose sugars, other minor sources of MG formation also exist and include catabolism of acetone and threonine [3,4]. At the same time, prokaryotes demonstrate the enzyme-catalyzed formation of MG as well, which takes place via the dephosphorylation of dihydroxyacetone phosphate (DHAP) [1,2]. These biomolecular modifications due to reactivity of MG leads to changes in the redox status of the cellular milieu. Since the generation of MG in the cell is inevitable, almost all organisms possess glyoxalase enzymes that restrict increase in MG levels in their cellular milieu and hence, contribute to redox homeostasis [5,6].

**Figure 1:**
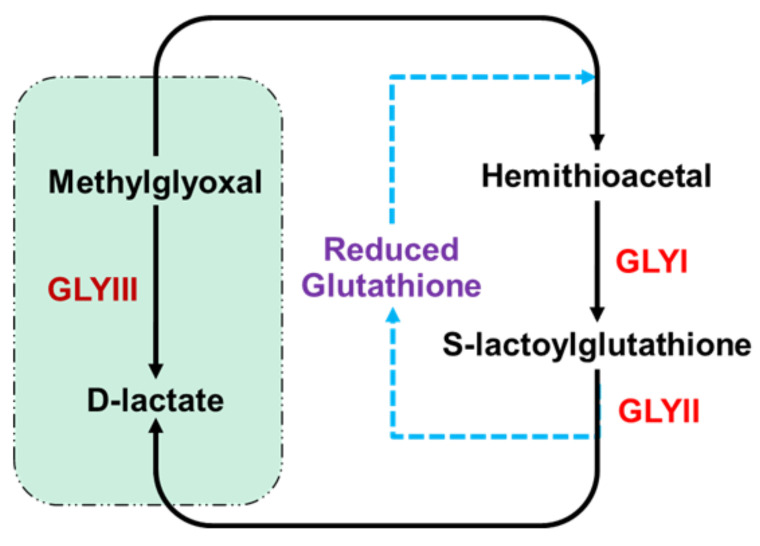
The glyoxalase pathway for metabolism of methylglyoxal. The classical two-step glyoxalase pathway converts methylglyoxal (MG) to D-lactate with the help of Glyoxalase I (GLYI) and Glyoxalase II (GLYII) enzymes and uses reduced glutathione (GSH) as cofactor. Glyoxalase III (GLYIII) constitutes the shorter pathway of MG detoxification which directly converts MG to D-lactate in a single step, without involving GSH.

Based on the glutathione (GSH) requirement, glyoxalases were categorized into two types, i.e., the GSH-dependent and the GSH-independent form [7,8,9]. The GSH-dependent system is a two-enzyme pathway involving GLYI and GLYII enzymes [10], whereas the GSH-independent system involves a single glyoxalase III (GLYIII) enzyme [7,8,9]. The metalloenzyme GLYI (requiring either Ni^2+^ or Zn^2+^) converts hemithioacetal formed from the spontaneous combination of MG and GSH to R-(S)-lactoylglutathione [11,12], which is further hydrolyzed by GLYII to give D-lactate and GSH [13]. On the other hand, GLYIII enzymes catalyze the direct conversion of MG to D-lactate without requiring GSH [7,8,9] (Figure 1).

While the GSH-dependent glyoxalase pathway is highly specific for the detoxification of MG and other dicarbonyls, the GSH-independent GLYIII proteins exhibit varied catalytic functions. These proteins possess the DJ-1_PfpI domains and belong to the DJ-1 enzyme superfamily, the members of which in addition to the GLYIII activity, exhibit various other physiological functions, such as the protease [14], chaperone [15], deglycase [16,17], esterase [18], anti-oxidant stimulant [19], mitochondrial regulation, and transcriptional regulator [20] functions. On similar lines, the human DJ-1 protein was reported to show protease and chaperone functions along with the GLYIII activity [20]. Besides, it also acts as a sensor of oxidative stress and is a major determinant for the early onset of Parkinson’s disease, with its loss leading to neurodegeneration [21]. In plants, the role of GLYIII proteins largely remains unexplored, with a few studies demonstrating their role in response towards environmental stresses [22] and chloroplast development [23]. Overexpression of DJ-1 (Hsp31) protein from *E. coli* was shown to confer tolerance against biotic as well as abiotic stresses in tobacco [24]. Similarly, ectopic expression of DJ-1_PfpI domain containing GLYIII from *Erianthus arundinaceus*, a wild-relative of sugarcane confers drought tolerance in commercially cultivated sugarcane hybrid [25]. With respect to their glyoxalase activity, it was reported that the GLYIII proteins have lower catalytic efficiencies as compared to the conventional GSH-dependent GLY pathway [9] but are particularly important under conditions of diminished GLYI/GLYII activity as a result of low GSH levels, such as in chronic oxidative stress, stationary phase, or sulfur limitation [26]. In addition to possessing various catalytic functions, the varied sub-cellular localization of GLYIII proteins makes them even more versatile and functionally diverse [9]. Hence, GLYIII proteins appear to be important for the normal functioning of living systems.

The availability of completely sequenced genomes of many organisms has allowed comparative analysis of various gene families across species, giving an insight into how the evolution and diversification of a protein family can help organisms evolve, as conditions change. One of our recent studies highlighted the expansion of glyoxalase family, including GLYIII, exclusively in plants and established the lack of motility as the major reason for the multiplicity of the glyoxalase gene family in plants [27]. The sessility of plants makes them more vulnerable to the different kind of stresses, and hence, these multigenic glyoxalase families provide redundancy in gene function and act as a contingency under extreme conditions [27]. Another study unraveled interesting information on the evolutionary trajectory of GLYI proteins, tracing the origin of the two metal-dependent forms to different prokaryotic ancestors along with elucidating the components of the plant GLYI family [6]. Since the discovery of GLYIII pathway raises many questions regarding the defense strategy of plants against MG stress, it will be worth investigating the distribution and evolution of these proteins across the different species. While the prokaryotes have just one catalytic (DJ-1_PfpI) domain [28], plant genomes possess GLYIII proteins with one or two catalytic domains [28]. How and when did the two-domain GLYIII proteins come into existence? How can the different domain architecture of these proteins be correlated to their diverse functions? These are the few questions that the present study aims to address via an investigation of the evolutionary trajectory of GLYIII proteins along with deciphering their structural and functional diversity in the plant kingdom.

## 2. Materials and Methods

### 2.1. Data Retrieval and Sequence Analysis

To extract amino acid sequences of GLYIII proteins from different organisms, either the conserved DJ-1_PfpI domain, PF01965 [29] or the sequence of HsDJ1-1 (BAG34938.1) and ScDJ-1 (EWG86848.1) proteins, was used as query. The NCBI database [30] was used to retrieve protein sequences for archaea, bacteria, protists, fungi, and animals using HsDJ1-1 and ScDJ-1 proteins as a query, while the Phytozome database [31] was used to retrieve plant DJ-1 proteins using Hidden Markov Model (HMM) profile search for the conserved DJ-1_PfpI domain. BLAST was done using the default settings. All the retrieved sequences were screened using SMART [32,33] and Pfam [29] for the presence of DJ-1_PfpI and other domains.

A total of 69 species were selected to cover the entire tree of life (representing five kingdoms, covering sub-divisions as well), and subsequently, GLYIII proteins from those species were used for further analysis, similar to that done for GLYI proteins [6]. The amino acid sequences of GLYIII used in the study are given in File S1 in Supplementary Materials. Sequences were aligned using ClustalW in MEGA 7.0 [34] with default settings.

### 2.2. Phylogenetic and Subcellular Localization Analysis

Phylogenetic trees were constructed using the amino acid sequence of the full-length GLYIII proteins or the corresponding domains. The sequences were aligned using MUSCLE [35], and subsequently, Maximum Likelihood tree was constructed using MEGA 7.0 [34] with default settings and 1000 bootstrap replicates. The tree was visualized and modified in iTol [36]. Subcellular localization of the proteins was predicted using online servers, CELLO [37,38] and WolfpSORT [39].

### 2.3. Motif and Three-Dimensional Structural Analysis of GLYIII Proteins

The plant GLYIII protein sequences were analyzed for the presence of conserved motifs using the MEME software suite [40]. Secondary structure predictions were made using the ESPript 3.0 program [41]. For homology modelling studies, SWISS-MODEL [42] tool of the Expasy server was used and then visualized in Pymol software [43].

## 3. Results

### 3.1. GLYIII Proteins Contain DJ-1_Pfp1 Domain in Combination with Various Additional Domains

**Figure 2:**
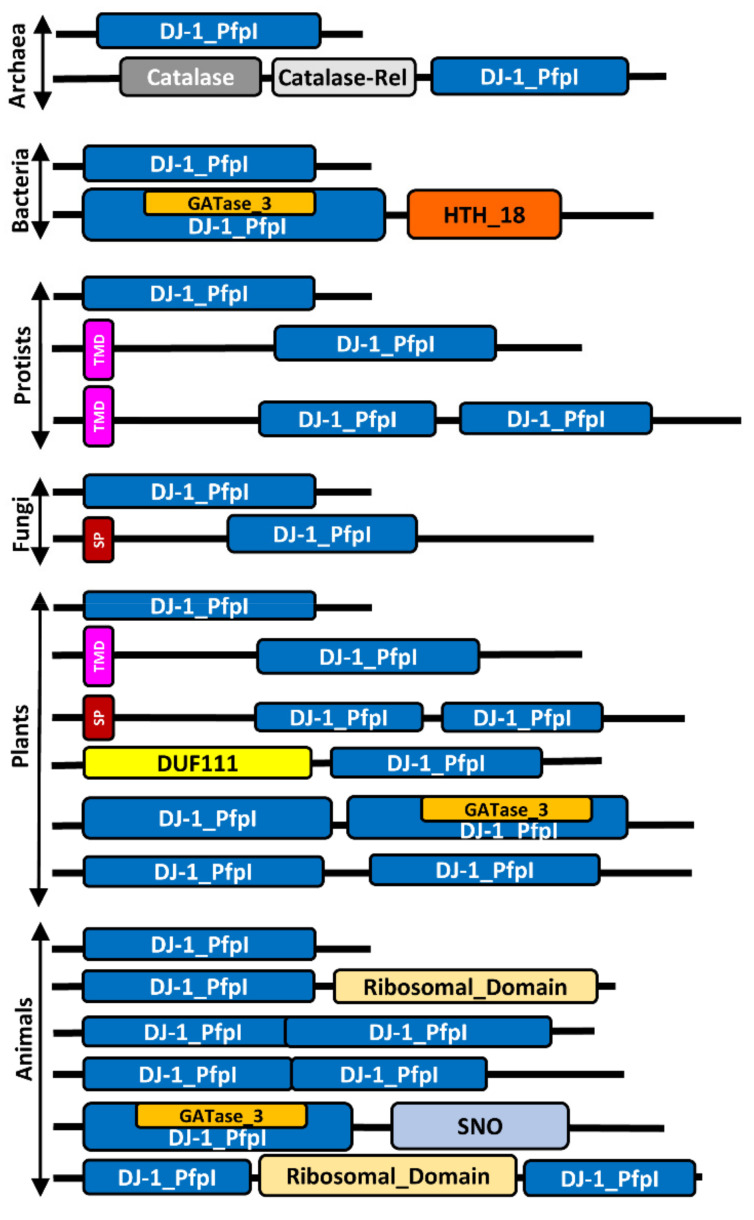
Schematic depiction of domain architecture observed in GLYIII proteins from different kingdoms. GLYIII proteins essentially contain DJ-1_PfpI domains often in combination with other domains such as catalase, HTH_18 (helix-turn-helix), DUF (Domain of unknown function), SNO (glutamine amidotransferase), etc. Two DJ-1_PfpI domains are common to plant GLYIII and some instance of its presence is also seen in animals. Image shown is not to scale. TMD—transmembrane domain, SP—signal peptide.

GLYIII protein sequences were extracted from 69 species representing different phyla to cover all the five kingdoms (Table S1). All GLYIII proteins possessed the hallmark DJ-1_PfpI domain, belonging to the respective superfamily (Figure 2). Besides, other domains are present in these proteins that may confer diverse functional properties (Figure 2). Of the five archaeal species used in the study, *Natronococcus occultus* was found to possess a catalase (PF00199) and a catalase-rel (catalase-related, PF06628) domain in addition to the DJ-1_PfpI domain (Table S2 in Supplementary Materials). These domains are present in catalase enzymes that protect the cells from toxic effects of hydrogen peroxide [44]. The catalase-rel domain additionally carries the immune-responsive amphipathic octa-peptide [45]. Interestingly, this catalase/catalase-rel domain was not observed in the GLYIII proteins from other kingdoms used in this study. Likewise, domain analysis of the bacterial GLYIII proteins revealed the presence of additional HTH_18 (PF12833) domain in about 9 of the 45 analyzed bacterial GLYIII proteins (Table S2 in Supplementary Materials). Of the 9 GLYIII proteins having this additional HTH_18 domain, six belonged to *Pseudomonas* sp. GM30 strain, which had a total of 11 GLYIII proteins, the other three belonged to two different bacterial species viz. *Rhizobium* sp. CF080 and *Desulfovibrio alkalitolerans* (Table S2 in Supplementary Materials). The helix-turn-helix (HTH) domain is a major structural motif capable of binding DNA, indicating the ability of such proteins to interact with nucleic acids as well. In contrast, GLYIII proteins from protists do not possess additional catalytic domains besides the DJ-1_PfpI domain. Although few protist GLYIII proteins have an N-terminal transmembrane domain required for their association with membranes, but may not have any additional specific catalytic functions (Figure 2). Importantly, the first instance of the presence of two DJ-1_PfpI domains could be seen in this kingdom as observed for *Dictyostelium discoideum* where in, it possessed two domains with unusually shorter (83 and 97 aa) lengths (Table S2 in Supplementary Materials). On the other hand, fungal GLYIII proteins which were thought to be evolutionary more advanced than the protists, had only single DJ-1_PfpI domain (Figure 2). However, they had a signal peptide sequence at the N-terminus, in some of the proteins.

In plants, GLYIII proteins majorly existed as two DJ-1_PfpI domain-containing proteins, while some proteins showed only single domain. In addition, some GLYIII proteins (both single as well as double DJ-1_PfpI domain-containing proteins) had a signal peptide or transmembrane domain at the N-terminus similar to that found in protists and fungi. In fact, a DUF111 (PF01969) domain was also found to coexist with DJ-1_PfpI domains as observed in PpGLYIII6 (Table S2 in Supplementary Materials). The presence of single DJ-1_PfpI domain-containing GLYIII proteins was, however, limited to some species, and that the majority of instances being of the dicots, the only exception in monocots being *T. aestivum* (Table S2 in Supplementary Materials). The green algae, *Chlamydomonas reinhardtii* and *Chlorella variabilis*, had only single DJ-1_PfpI domain-containing GLYIII proteins, whereas the moss *Physcomitrella patens,* which is the ancestor of land plants, had both single and double DJ-1_PfpI domain-containing GLYIII proteins. Further, like plants, animals also possessed both single and double DJ-1_PfpI domain-containing GLYIII proteins; however, with a predominance of single-domain proteins (Table S2 in Supplementary Materials). The two domains in case of double DJ-1_PfpI domain-containing proteins as in *Echinops telfairi* (EtGLYIII2) and *Tupaia chinensis* (TchGLYIII3), had domains that are either overlapping or conjoint. Some animal GLYIII proteins harbored SNO (PF01174) or Ribosomal (Ribosomal_L18_c, PF14204 or Ribososmal_L16, PF00252) domains, the latter having implications in rRNA binding (Figure 2). Notably, some species in bacteria, animals, and even plants possessed GATase3 domain, representing a CobB/CobQ-like glutamine amidotransferase domain, enclosed within the DJ-1_PfpI domain (Figure 2). The GATase3 domain possesses similar catalytic sites as found in DJ-1 proteins, which is the reason for the observed overlap of the two domains.

Further, we found the length of the DJ-1_PfpI domain in GLYIII proteins ranged from 52 to 282 amino acids (Table S1). The shortest domain (52 aa) was observed in *T. aestivum* TaGLYIII4 and the longest (282 aa) in *E. coli* EcGLYIII1 (Table S1). However, the average domain size was about ~162 aa. Though variable in length, the GLYIII domain in plants, based on the domain lengths were majorly of two types, smaller ones, which were approximately 165 aa in length and the larger ones which were about 184 aa long. However, there was no particular pattern of inheritance of these domains in the plant GLYIII family, but the domains of similar size usually co-existed in the two DJ-1_PfpI domain-containing GLYIII proteins (Table S1). In contrast, plant GLYIII proteins having single DJ-1_PfpI domains, generally had very short domain lengths (55–146 aa). Similarly, animal GLYIII proteins having two DJ-1_PfpI domains also had both the domains being very short in length (52–90 aa). But considering the general trend, even animal GLYIII proteins like plants had shorter (165 aa) as well as longer (184 aa) type of domains.

### 3.2. Plants Have Three Major Types of GLYIII Proteins

**Figure 3:**
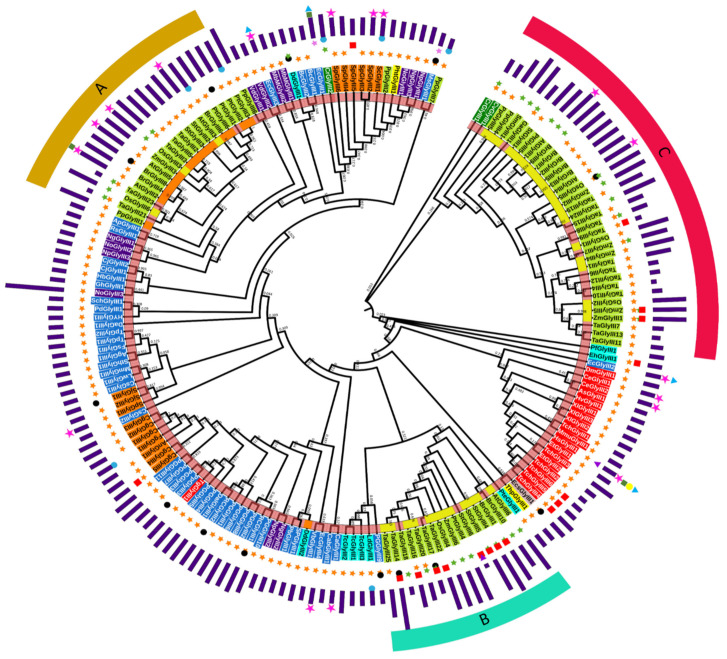
Phylogenetic analysis reveals three types of GLYIII proteins exist in plants. In all, 183 sequences from 69 species were used. The tree indicates the presence of three types of plant GLYIII proteins represented as cluster-A (mustard strip), B (pale-green strip), and C (red strip). Innermost strip indicates the length of linker region separating the two domains. Yellow-green color represents longer linker length (37–39 aa), orange indicates small linker length (9–20 aa), and light coral indicates no linker region. Purple bars on the outer side of the tree indicate protein length. Symbols next to protein names indicate different organellar localization patterns. Orange star indicates cytoplasmic localization, green star- chloroplast, purple star—periplasm, black circle-membrane (inner and outer membrane for prokaryotes, and plasma membrane for eukaryotes), blue circle—extracellular, red square—mitochondria, and purple triangle—nuclear localization. GLYIII proteins belonging to the different phylogenetic domains of life are depicted using specific colors as, archaea (purple), bacteria (blue), protists (cyan), fungi (orange), diatoms (yellow), brown algae (light brown), green algae (dark green), plants (light green), and animals (red). The different colored symbols above the bars (representing protein size) indicate different biochemical activities, i.e., GLYIII (pink star), chaperone (green square), protease (yellow circle), and deglycase (blue triangle). The maximum likelihood tree has been constructed with 1000 bootstrap replicates (indicated over branches) using MEGA-7.0 and visualized using iTOL.

To understand the evolutionary relationship of GLYIII proteins from different organisms, a maximum likelihood tree was constructed using ClustalW in MEGA7.0 (Figure 3). Of these, only some proteins have been biochemically characterized and same is indicated in the tree (Figure 3). Through this protein-based phylogenetic analysis, we acquired initial indications on GLYIII protein relationship among different organisms, simultaneously correlating them with their biochemical and molecular features. Further, we determined whether any prokaryotic or eukaryotic GLYIII had the same composition of domains as plant GLYIII, in turn revealing conservation among these proteins. We found, besides plants, several other organisms also possessed multiple GLYIII proteins. The tree clearly showed three major types of plant GLYIII proteins, one closely grouped with the prokaryotic homologs (named as cluster-A), the other (cluster-B) having a closer relationship with the animal and protist GLYIII proteins, and the third (cluster-C) forming a distinct group, not clustering with proteins from any other kingdom (Figure 3). All the three clusters contained single as well as double DJ-1_PfpI domain-containing GLYIII proteins, with proteins from monocots and dicots forming separate sub-clusters within each cluster. However, no significant difference was found between monocot and dicot GLYIII proteins.

Further, GLYIII members of these three clusters were generally longer in length than those from the other organisms due to the presence of two DJ-1_PfpI domains in them as against the rest which possesses single DJ-1_PfpI domain. Moreover, cluster A, B, and C proteins also possessed an additional stretch of amino acids known as the linker region, which connect the two domains (Figure 3; Table S1). Interestingly, the two DJ-1_PfpI domain-containing GLYIII proteins in cluster-A had shorter linker regions (9–20 aa), except for AtGLYIII3, OsGLYIII3, and OsGLYIII6 proteins, while the cluster-B and cluster–C proteins had longer linker regions of approx. 37–39 aa long, as shown in Figure 3. Further, the proteins with longer linker regions had relatively shorter domain lengths (~165 aa for both the domains), while the proteins with shorter linker regions had longer domain lengths (~185 aa for both domains) (Figure 3; Table S1), indicating a tight correlation between the length of the linker region and the domain size.

Another important observation pertains to the presence of GLYIII proteins from green algae in cluster-C of the tree (Figure 3). The results clearly indicated that proteins of the cluster-C represent a plant-specific lineage derived from their green algae ancestors. Further, cluster-B proteins were observed to be a part of the larger cluster comprising of proteins from protists, brown algae, diatoms, and animals, which suggests that members of this cluster have undertaken a vertical path during the course of evolution. On the other hand, cluster-A proteins belonged to a larger cluster comprising majorly of prokaryotic GLYIII, especially grouping with archaeal and bacterial GLYIII proteins with no closer eukaryotic homolog being identified. It is possible that horizontal gene transfer events have shaped their evolution or cluster-A type of proteins were lost or diverged in animals.

### 3.3. Cluster-C GLYIII Proteins Share Similarity with Their Ancestral Homologs in Localization Patterns

After gaining initial insights into the evolution of plant GLYIII proteins from their prokaryotic ancestors, we next investigated their subcellular localization patterns to further unravel the evolutionary path of these proteins. The predictions for subcellular localization were made using CELLO [37,38] and WolfpSORT [39]. In prokaryotes, GLYIII proteins were predicted to be generally cytoplasmic, except for very few species, which were predicted to be either extracellular, periplasmic, or membrane-localized (Figure 3). Likewise, our predictions indicated that GLYIII proteins from the kingdom Protists and fungi also have few extracellular, membrane-localized, or even mitochondria-localized isoforms. Further, the majority of the animal GLYIII proteins were predicted to be in the cytoplasm with the exceptions of few mitochondrial candidates.

In comparison to the other kingdoms, GLYIII proteins from plants exhibited greater variations in their localization properties, attributed primarily to the differential predicted localization patterns of the three plant-specific GLYIII clusters. For instance, most of the cluster-A proteins are possibly cytoplasmic except for OsGLYIII3, OsGLYIII6, and PtGLYIII1, which were predicted to be exclusively localized to the chloroplast. In fact, OsGLYIII3 and OsGLYIII6 formed exceptions even in terms of the linker region length, which was not similar to other members of this group, whereas PtGLYIII1 was a single DJ-1_PfpI domain-containing protein, indicating their inherent outlier nature (Figure 3). On the other hand, most of the cluster-B members were predicted to be chloroplast and/or mitochondria localized. Further, GLYIII proteins from green algal ancestors of plants, which were the members of cluster-C, were predicted to be localized to the cytoplasm and/or chloroplast and consequently, most of the members of this group were either cytoplasmic or chloroplastic, with very few (TaGLYIII9, ZmGLYIII1, and ZmGLYIII5) being predicted to co-localize in mitochondria as well. In agreement, GLYIII proteins from the moss, *P. patens*, which is an evolutionary intermediate between the green algae and the land plants, also exhibited putative cytoplasmic and chloroplastic localization of two of its members belonging to this cluster. In fact, it could be generalized that almost all plant species under investigation had at least two members in this cluster, one predicted to encode a cytoplasmic and another a chloroplastic isoform. Overall, assessment of sub-cellular localization suggested that plant GLYIII proteins of the three clusters had different evolutionary trajectories. In particular, proteins of cluster-C were more primitive, sharing their possible localization patterns with ancestral homologs from green algae and the moss *P. patens*.

### 3.4. Assessment of Domain Relationships of GLYIII Proteins Indicate Primitive Evolution of N-Terminal Domain of Plant GLYIII

**Figure 4:**
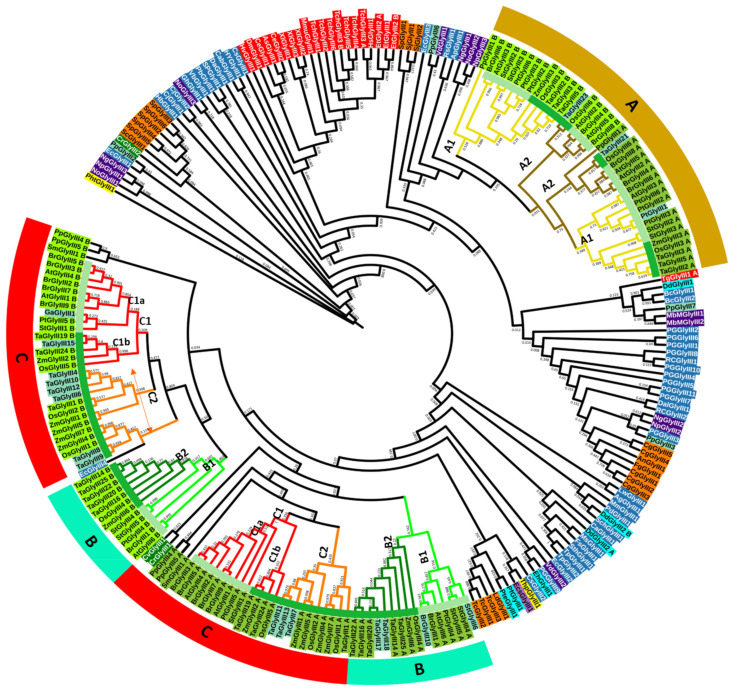
Domain relationship between GLYIII proteins unravel differential evolution of two DJ-1_PfpI domains in plants. The maximum likelihood tree was constructed with 1000 bootstrap replicates (indicated over branches) in MEGA 7.0 using 238 sequences from 183 GLYIII proteins belonging to 69 species. Different sub-clusters (A1&2, B1&2, C1&2) are indicated in the tree and nodes are colored differently to enable ease in understanding. The two domains of plant GLYIII proteins group separately in each cluster and can be identified from the suffix A (for first or N-terminal domain) and B (for second or C-terminal domain). Outermost strip indicates the three types of plant GLYIII proteins represented as cluster-A (mustard strip), B (pale-green strip), and C (red strip). Innermost strip indicates monocot (dark green) and dicot (pale green) species. GLYIII proteins belonging to the different phylogenetic domains of life are depicted using specific colors as, archaea (purple), bacteria (blue), protists (cyan), fungi (orange), diatoms (yellow), brown algae (dark orchid), green algae (dark green), and plants (A-domain: light green, B-domain: dark olive green, plants with single domain: aquamarine) and animals (red).

Since plant GLYIII possessed two DJ-1_PfpI domains, we analyzed the evolutionary trajectory of each domain. For this, phylogenetic relationships among the DJ-1_PfpI domain sequences from different species were investigated (Figure 4). Both the domains of plant GLYIII proteins formed two separate clusters, with first domain (or N-terminal domain, indicated by suffix ‘A’) of all proteins grouping together and the second or C-terminal domain (indicated by suffix ‘B’) forming a separate cluster. Importantly, proteins belonging to cluster-A had both the sub-clusters representing the two domains in the same major cluster-A, with both sub-clusters grouping to the same node (Figure 4). Whereas, the members of cluster-B and cluster-C collectively formed two clusters, representing the two domains, with each domain within cluster-B and C grouping to different nodes (Figure 4).

Each of the two groups representing the two domains in cluster-A could be further sub-grouped into two smaller clusters (A1 and A2), indicating further divergence in domain sequences of cluster-A proteins (Figure 4). Of the two sub-clusters A1 and A2, the bigger sub-cluster A1 comprised of AtGLYIII3 and OsGLYIII3 domains from Arabidopsis and rice, respectively, full-length proteins of which were previously shown to possess high catalytic efficiencies as GLYIII [9,46]. The other sub-cluster A2 comprised of AtGLYIII5, AtGLYIII2, OsGLYIII6, BrGLYIII4, and BrGLYIII8 proteins. Interestingly, GLYIII proteins carrying single DJ-1_Pfp1 domain from wheat, TaGLYIII21 (clustering with N-terminal domain) and TaGLYIII23 (clustering with C-terminal domain) also grouped in the sub-cluster A2. In fact, analyzing the domain sequence similarity of these plant GLYIII proteins with those of *E. coli* (where GLYIII proteins are well-characterized), revealed greater similarity of cluster-A domains with EcGLYIII3 and of cluster-B/C domains with EcGLYIII2 protein (Table S3 in Supplementary Materials). Few exceptions included AtGLYIII3, BrGLYIII6, and PtGLYIII6 proteins, which have one domain more similar to EcGLYIII2 and the other being closer to EcGLYIII3 in terms of sequence similarity. Further, multiple sequence alignments of plant GLYIII proteins also uncovered the presence of an extra stretch of 4–18 aa in the domains of cluster-A proteins, the same being observed in both the domains of proteins belonging to this cluster (Figure S1 in Supplementary Materials). Another interesting observation pertained to the PpGLYIII1 protein, whose two domains grouped in different sub-clusters of the bigger cluster-A. Its N-terminal domain (or PpGLYIII1_A) grouped with N-terminal GLYIII domain sequences of cluster-A2, whereas the C-terminal domain (PpGLYIII1_B) grouped within the C-terminal GLYIII domains of cluster-A1, indicating differential origins of the two domains of cluster-A proteins.

Similar to cluster-A, the N- and C- terminal domains of GLYIII proteins in cluster-B and cluster-C, also formed separate sub-clusters. Importantly, GLYIII proteins from green algae which possessed single GLYIII domains were more similar to N-terminal domain of GLYIII proteins of cluster-C. Notably, both N and C terminal domains of GLYIII proteins belonging to cluster-B and cluster-C can be further subdivided into smaller clusters, B1-B2 and C1-C2, respectively, much like that observed for cluster-A proteins (Figure 4). The sub-clusters B1 and B2 of the main cluster-B consisted of GLYIII proteins from dicots and monocots, respectively. Similar grouping can also be observed in cluster-C where cluster C1a and C1b of the sub-cluster-C1 comprised of dicot and monocot GLYIII proteins, respectively. In addition, we observed another cluster in the major cluster-C, which is named as C2, comprising of proteins from only monocots (Figure 4). Importantly, this differential clustering of GLYIII from monocots and dicots was also observed in cluster-A proteins, with both the sub-clusters A1 and A2 being sub-divided into groups containing proteins from either monocots or dicots. Notably, GLYIII domains from the green algae grouped with the N-terminal domain sequences of GLYIII proteins, which suggested that the N-terminal domain of the two-domain GLYIII may have evolved first, which may have subsequently given rise to the C-terminal domain through gene duplication events.

### 3.5. Extensive Motif Rearrangements Have Shaped the Evolution of Plant GLYIII Proteins

**Figure 5:**
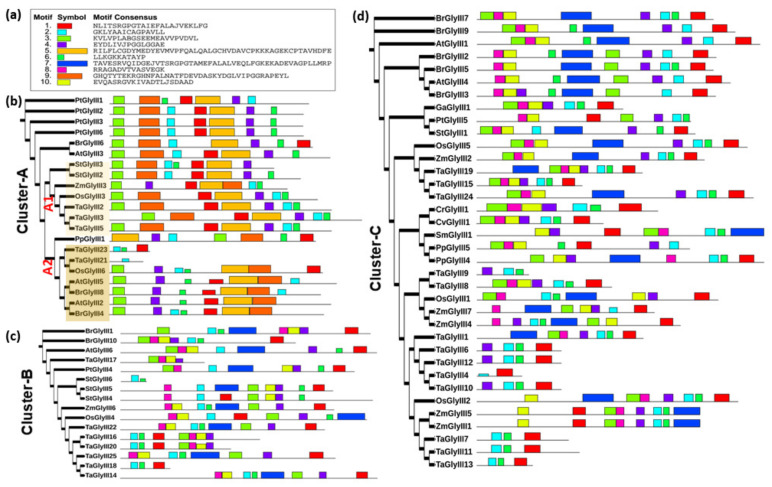
Extensive motif rearrangements have occurred during evolution of plant GLYIII proteins. (**a**) List of conserved motifs predicted in plant GLYIII proteins. Block diagram depicting motif organization in (**b**) Cluster-A, (**c**) Cluster-B, and (**d**) Cluster-C proteins. Conserved motifs in 73 GLYIII protein sequences from different plant species and green algae were predicted using MEME suite.

The plant GLYIII proteins are found to be of three different types. Hence, to further understand the evolutionary differences between them, we analyzed the presence of conserved motifs using the MEME suite. In all, ten conserved motifs were predicted upon analysis of 73 GLYIII sequences from different plants and green algae (Figure 5a). It was evident from the assessment that proteins in different clusters varied with respect to both, presence and arrangement of motifs (Figure 5b–d). Assessment of catalytic triad (Cys-His-Asp/Glu) in these proteins also suggested differences in residue conservation among these clusters (Figure S2). Of the three clusters (A, B and C), cluster-A proteins comprising of about seven motifs (3-9-2-1-5-4-6), showed least variations in comparison to the other two cluster proteins (Figure 5b). In agreement, the catalytic triad was highly conserved in cluster-A proteins, with both the domains of most of the cluster-A proteins showing complete conservation of residues (Figure S2). Importantly, proteins belonging to this cluster had ‘His’ residue essentially conserved in the first domain, with majority conserving ‘His’ even in second DJ-1_PfpI domain. As against cluster-B and -C, proteins in cluster-A lacked motifs 7, 8, and 10 and instead possessed motifs 5 and 9, which were unique to its members, not detected in group B and C proteins (Figure 5b–d). Closer examination of motif arrangements revealed that the two sub-clusters of the cluster-A also exhibited differences in motifs. The dicot members of the cluster A1 possessed 7 motifs majorly in the order 3-9-2-1-5-4-6, whereas monocot members contained these domains in the order 3-9-1-5-4-2-6 (Figure 5b). The PpGLYIII1 protein is an interesting case, wherein it contained all seven motifs; however, in a different order, with N- and C-terminal motifs being swapped in position (5-4-6-3-9-2-1). That was the reason we observed grouping of both domains of PpGLYIII1 in a different than usual manner (Figure 5). On the other hand, the smaller cluster A2 possessed motifs largely in the order, 3-4-2-1-5-9, with motif 6 being flexible in its location in these proteins (Figure 5b). Further, in comparison to the cluster-A, cluster B and C proteins were disorganized with no predominant pattern being observed (Figure 5c,d). In particular, most of the variations were due to the monocot members of these clusters. Further, as some of the *T. aestivum* GLYIII proteins were single-domain proteins, it was a further source of observed variations in the motif organization pattern. Assessment of triad residues revealed cluster-B and -C proteins to lack conserved ‘His’ from the triad in both the domains and ‘Cys’ being absent from the second domain of cluster-B proteins (Figure S2). However, it is to be noted here that these predictions were based on mere sequence alignment and that modelling of proteins may give more accurate prediction of presence/absence of active site residues.

### 3.6. Assessment of Motif Organization in DJ-1_PfpI Domains Reveals Both Vertical and Horizontal Evolutionary Patterns of Plant GLYIII Domains

**Figure 6:**
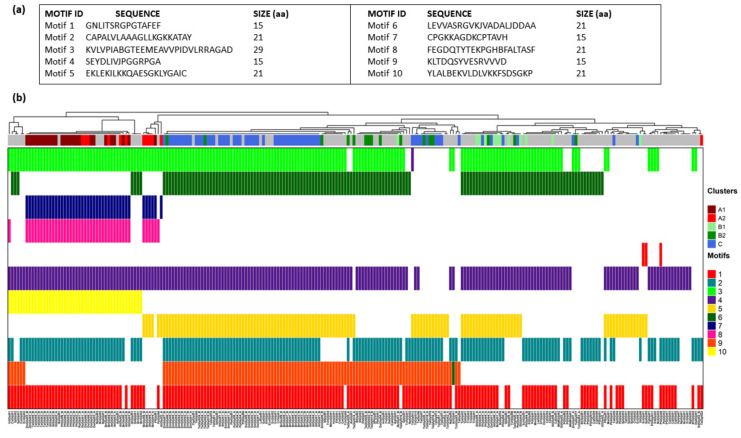
Motif organization pattern of domain sequences of GLYIII proteins from different organisms reveal distinct evolutionary trajectory of plant GLYIII clusters. (**a**) List of conserved motifs predicted in the domain sequences of GLYIII proteins from 69 species. (**b**) Heatmap showing distribution of motifs in domain sequences of all organisms under study. A total of ten motifs were identified in different combinations as depicted by different colors. Color strip on the top indicates clusters to which respective proteins belong. Gray indicates proteins not being clustered into either of the three Clusters- A (A1 and A2), B (B1 and B2), or C. MEME suite was used for prediction of motifs.

To understand the origin of individual DJ-1_PfpI domains, we analyzed the motif organization in domains of all the 183 GLYIII proteins (with either one or two domains) under study (Figure 6; Table S4 in Supplementary Materials). In all, 238 domain sequences were used to derive conserved patterns, which gave clear indications regarding domain evolution and also conveyed differences among the three plant GLYIII-specific clusters. Importantly, we analyzed ten motifs in all (which are different from those obtained while analyzing complete protein sequences) (Figure 6a). GLYIII proteins in the cluster-A possessed 7 motifs (3-7-8-4-10-2-1), which were also conserved in archaeal species like *Natronococcus occultus* (NoGLYIII2), *Natrinema pellirubrum* (NpGLYIII3), and *Natronobacterium gregoryi* (NgGLYIII1), and even in bacteria as *Acetobacter pasteurianus* and *Ralstonia solanacearum* (Figure 6b). Both the domains of proteins belonging to cluster-A possessed this seven-motif pattern with few exceptions, especially in cluster-A2. Herein, the first domain of AtGLYIII2/5 and BrGLYIII4 and both the domains of BrGLYIII8 showed a different pattern of motif organization viz. 3-7-8-4-5. Other exceptions pertained to the absence of one or more motifs, the loss of motif 2 being the most common exception in cluster-A2 proteins. Further, members of cluster B (B1 and B2) and C shared the same motif composition and organization, in the form of seven motifs arranged in the order, 3-6-4-5-2-9-1. The larger cluster-C was, however, more consistent in the presence of this motif pattern, while the cluster-B proteins mostly showed absence of either of the motifs from the 4-5-2-9 motif combination. Compared to cluster-A, in cluster-B/C, a single motif 6 replaced the two cluster-A motifs, 7 and 8. Moreover, motifs 5 and 9 were unique to cluster -B and –C members, with the exception of few cluster-A2 members, which also possessed motif 5, as discussed above. Usually, members of cluster-B1 possessed motif 5, whereas those of cluster-B2 possessed motif 9. The general motif pattern of cluster-B/C proteins was also common to GLYIII proteins from some bacteria (as *Pseudomonas sp.* GM30, PGGLYIII9 and *E. coli*, EcGLYIII2), diatom (like *Thalassiosira pseudonana* CCMP1335, ThpGLYIII1), brown algae (*Ectocarpus siliculosus*, EcsGLYIII1), and protists (*Entamoeba histolytica*, EhGLYIII1 and *Leishmania donovani*, LdGLYIII1), which also inherited these motifs (Figure 6b). However, animal GLYIII despite sharing close sequence similarity with the cluster-B proteins, possessed certain differences with respect to the presence of motifs 2 and 5. As against plant GLYIII, which possessed seven motifs, animal GLYIII has six motifs, though in essentially the same order as of cluster-B proteins, but either motif 2 or motif 5 was absent. Overall, the results indicated that cluster-B and -C members have been probably inherited from prokaryotes through vertical descent as we observed the similar motif architectures in several prokaryotes, plants, and animals. In contrast, cluster-A type of motif organization was restricted to archaeal and few bacterial species along with plants, indicating cluster-A2 proteins have different ancestry than that of cluster B/C proteins and probably have been acquired through horizontal gene transfer events in plants.

### 3.7. Variations in the Three-Dimensional Predicted Structure of Plant GLYIII Proteins from Different Clusters Are Indicative of Their Functional Flexibility

**Figure 7:**
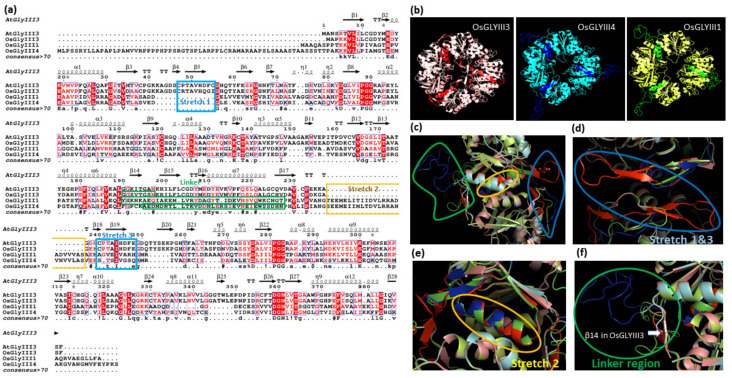
Structural differences in rice GLYIII proteins belonging to three clusters are indicative of their functional flexibility. (**a**) Sequence alignment and secondary structure information of rice OsGLYIII3, OsGLYIII4, and OsGLYIII1 proteins using ESpript3.0. (**b**) Homotrimeric structure of OsGLYIII3, 4 and 1 as modelled using SWISS-MODELLER. (**c**) Enlarged view showing regions of structural dissimilarity between the three proteins. Enlarged view showing region corresponding to (**d**) stretch 1 and 3, (**e**) stretch 2 and (**f**) linker region, in the aligned monomers of the three proteins. AtGLYIII3 was used as the template for both secondary and tertiary structure predictions. Structures were visualized in PYMOL.

Following the assessment of domain and motif organization, we examined the secondary and three-dimensional (3D) structures of the plant GLYIII proteins. For this, GLYIII proteins from rice viz. OsGLYIII3, OsGLYIII4, and OsGLYIII1 were selected, which served as representatives of clusters A, B, and C, respectively. These were initially analyzed for their secondary structure using ESPript 3.0 program (Figure 7a). Subsequently, 3D structures were modelled based on homology using SWISS-MODEL (Figure 7b–f). For both secondary structure prediction and 3D structure modelling, AtDJ-1D protein (PDB ID: 3uk7.1.A) from Arabidopsis was used as the template. The predicted structures were obtained as homo-trimers (Figure 7b) in line with the similar oligomeric nature of the AtDJ-1D protein. An overall high structural similarity could be observed between the three GLYIII proteins (Figure 7b).

Secondary structure alignment revealed the presence of several α-helices (12), β-strands (28), and 3_10_-helices (9) in the GLYIII proteins with a predominance of β-strands. In addition, strict β-turns (TT) were also predicted in the polypeptide chains (Figure 7a). There were certain regions of difference between OsGLYIII3 (belonging to cluster-A) and cluster-B (OsGLYIII4) and -C (OsGLYIII1) members. For instance, the region between residues 40 and 50 corresponding to β4 and β5 strands (marked by a blue box; stretch 1) was specific to OsGLYIII3 and not present in the other two members. However, a similar motif in the C-terminal domain (represented by β18 and β19 strands and enclosed in a blue box; stretch 3), was found to be present in all the three proteins. The other significant difference was in the region marked by a yellow box (stretch 2), which was not found in OsGLYIII3, but was present in the other two proteins (Figure 7a,c).

Inspection of these regions of difference in the 3D structure, however, revealed several discrepancies (Figure 7c). While the stretch 1 was predicted not to be present in OsGLYIII1/4, no overlay corresponding to OsGLYIII1/4 was observed even in stretch 3 as against the predicted secondary structure alignment. The regions enclosed in a blue circle (Figure 7c) represents the β-strand encoded by the stretch 1 and 3 residues in OsGLYIII3, enlarged view of which can be observed as red color β-strands (Figure 7d). Further, the stretch 2, which was predicted to be absent in OsGLYIII3 (Figure 7a) was, however, found to be aligned as an α-helix structure, with the other two proteins (Figure 7c,e). Interestingly, linker regions marked in green boxes (Figure 7a) did not align in the overlay of OsGLYIII1 and OsGLYIII4 (Figure 7f). The linker region was generally, shorter in the cluster-A proteins, to which OsGLYIII3 belonged, and in fact, existed as a β-strand (β14) in OsGLYIII3 (Figure 7f). Altogether, these three proteins possessed higher overall similarity in structures, but regions of distinctiveness in their structures may offer scope for functional diversity in GLYIII proteins.

## 4. Discussion

GLYIII enzymes belong to the DJ-1_PfpI superfamily, with its members mediating diverse functions such as proteases [14] and chaperones [15]. The human DJ-1 protein, one of the most extensively studied members of this superfamily, was reported as the genetic cause of early-onset of Parkinson’s disease and found to exhibit weak protease activity, chaperone functions, and even glyoxalase activity [14,15,16]. Importantly, members of this superfamily possess a catalytic triad, Cys-His-Asp/Glu, known to be required for its catalytic activity [47]. The cysteine residue is functionally essential, being invariantly conserved in all the members of this superfamily and can participate in different functions owing to the presence of a reactive thiol group. The requirement of cysteine was essentially realized in the reaction chemistry of different functions viz. GLYIII, chaperone and protease activities [14,15,16] and may even act as a sensor of cellular redox homeostasis [48].

Assessing the distribution of DJ-1_PfpI (or GLYIII or DJ-1) proteins across different kingdoms revealed their ubiquitous presence across the tree of life, much like the GLYI and GLYII proteins [6,28]. Interestingly, multiple DJ-1 isoforms are found to be present in all kingdoms and not specifically restricted to plants, as otherwise is the case for GLYI/II genes, which have specifically expanded in plants. Nonetheless, our results suggest that plants do possess more copies of DJ-1 genes than the other species. The presence of multiple DJ-1 in most phyla is possible because, proteins belonging to the DJ-1 superfamily are functionally diverse, performing various essential functions encoded by different members, contrary to the role of GLYI/II proteins, which have a highly specific catalytic function in MG detoxification. Another similar feature of plant GLYIII (or DJ-1) with that of the plant GLYI (Ni-GLYI) is the evolution of two catalytic domain-containing DJ-1 proteins, although single DJ-1 domain-containing proteins are also detected in some plants, especially monocots. In agreement, single domain GLYI also exists in plants that are the Zn/Mn dependent enzymes [6].

The first instance of the presence of two DJ-1 domains in a single polypeptide is seen in the protist *Dictyostelium discoideum* (DdGLYIII2); however, these domains are different from the typical domains, being smaller in length ranging from about 83–97 aa. The first domain has the motif composition 3-6-4, and the second or C-terminal domain contains only two motifs 2-1. This is in sharp contrast to the seven-motif composition of plant GLYIII domains (3-6-4-5-2-9-1). Presence of such DJ-1 protein appears more as an exception, as all the analyzed algal species possess only single DJ-1 domain-containing proteins. Further, the moss *P. patens*, a non-vascular ancestor of land plants, is the first species to harbor both single and two-domain proteins and thereafter, the two DJ-1_PfpI domain-containing proteins can be observed in the other plant lineages. In addition to the DJ-1_PfpI domains, members of this superfamily generally possess other catalytic domains, as well such as a catalase domain in archaea *Natronococcus occultus* or the HTH_18 domain in the bacteria *Pseudomonas* sp. GM30, which may add functional diversity or facilitate the regulation of these proteins. Even the human DJ-1 protein was shown to bind multiple RNA targets in an oxidation-dependent manner [49].

Analysis of phylogenetic relationship among DJ-1 proteins from different kingdoms uncovers an interesting evolutionary path, much different from that observed for the GLYI proteins [6]. Our studies suggested that plant DJ-1 proteins have evolved into three different types of proteins, which we categorized into clusters A, B, and C, with all the clusters being further sub-grouped into smaller clusters. Since these proteins perform multiple functions, it is possible that divergence in sequence between the different clusters is related to the selectivity in functions of these proteins. Cluster-A was more closely related to prokaryotic DJ-1 proteins, being enclosed within the larger clade composed of prokaryotic and yeast DJ-1 proteins. The members of cluster-A had the typical domain motif patterns comprising of motifs 3-7-8-4-10-2-1. Of the four *E. coli* GLYIII (or DJ-1) proteins, two isoforms viz. EcGLYIII1 (or Hsp31), and EcGLYIII3 (or YhbO), along with yeast DJ-1 proteins SpGLYIII2-SpGLYIII6 (or Hsp3101-Hsp3105) from *S. pombe* and ScGLYIII1 (or Hsp31) from *S. cerevisiae* belong to this clade. However, except for a few archaeal GLYIII (NgGLYIII1, NoGLYIII2, and NpGLYIII3) and a bacterial GLYIII (RsGLYIII1), no other protein from this parent group was found to contain the cluster-A type of motif structure. On the other hand, cluster-B was positioned close to the animal DJ-1 proteins and possessed more similarity to the eukaryotic DJ-1. In fact, protists, brown algae, and diatoms also co-existed within the same larger cluster to which cluster-B belongs. Among the prokaryotes, the GLYIII protein YajL (EcGLYIII2) from *E. coli* shared close similarity to this cluster. Analyzing the motif organization of proteins from plants and the above-mentioned organisms revealed that the members possessed the motif pattern 3-6-4-5-2-9-1, with sub-groups B1 and B2 differing in the presence of motifs 9 and 5, respectively. Animal GLYIII members, however, possess either of the two motifs, 2 or 5, with the rest being the same. The absence of motif 2 in humans, was the main point of distinction between the plant and animal GLYIII sequences. In contrast, cluster-C was comprised exclusively of plant GLYIII proteins, not containing any members from other organisms and possessed the same motif organization pattern as of cluster-B proteins.

Assessment of functional properties of plant GLYIII proteins from the three clusters revealed differential catalytic features of the respective proteins. The OsGLYIII3 (or OsDJ-1c) from rice [9] and AtGLYIII3 (or AtDJ-1d) from Arabidopsis [46] belonging to cluster-A1, exhibited higher levels of GLYIII activity in comparison to the rest of the members of their family, which indicated that cluster-A1 comprised of catalytically more efficient GLYIII proteins. Likewise, EcGLYIII1 and EcGLYIII3 proteins from *E. coli* [50], ScGLYIII1 from *S. cerevisiae* [51], and SpGLYIII2-SpGLYIII6 from *S. pombe* [8] were also demonstrated to possess GLYIII activity. Similar to AtGLYIII3 (AtDJ-1d), all these members can use both MG and glyoxal (GO) as substrates and in fact, show higher activity with GO than MG. While the smaller cluster A2, comprising of proteins like AtGLYIII2 (AtDJ-1e) and AtGLYIII5 (AtDJ-1f), exhibited lower levels of MG-specific GLYIII activity and did not use GO as substrate [46]. Further, proteins in the cluster-C such as AtGLYIII1 (AtDJ1-b) and AtGLYIII4 (AtDJ1-a), were also active as GLYIII enzymes but possessed low levels of activity in comparison to the cluster-A1 representatives from Arabidopsis. Again, the activity of AtDJ1-a and AtDJ1-b was higher when GO but not MG, was used as the substrate [46], much like EcGLYIII3, ScGLYIII1, and SpGLYIII2-6 [8]. The AtDJ1-a protein is involved in the process of ageing, as the loss of its function resulting in accelerated cell death in aging plants [22]. It confers stress protection through cytosolic SOD1 (superoxide dismutase) activation. While AtDJ-1b possesses both holdase chaperone function and GLYIII activity, which is sensitive to H_2_O_2_ [15]. On the similar lines, EcGLYIII2 and EcGLYIII4 possess GO-specific GLYIII activity and are not able to utilize MG as the substrate [50]. On the other hand, AtGLYIII6 (or AtDJ-1c) in cluster B (specifically cluster-B1, when looking at the domain-specific phylogeny) did not exhibit GLYIII activity and was involved in chloroplast maturation [23], indicating functional divergence in such proteins [46].

Another important observation pertains to the observed high sequence similarity between the two domains of the two DJ-1 domain-containing proteins of cluster A1. The first or N-terminal domain shared more than 50% sequence identity to the second or the C-terminal domain, which was highly indicative of domain duplication followed by domain fusion events behind the emergence of the two-domain DJ-1 proteins in this cluster, whereas proteins in cluster A2 possessed about 30% sequence identity between the two domains. Likewise, less than 50% similarity was also seen between the two domains of each member of the clusters B and C, except for SmGLYIII1, PpGLYIII4, and PpGLYIII5 proteins, which shared 55–59% sequence identity between their domains. In fact, proteins of cluster A and B/C had some characteristic features specific to their groups. For instance, domains of cluster-A proteins were generally longer in length (~184 aa) than the cluster-B/C proteins (~165 aa), but had shorter linker regions (~9–20 aa) than these proteins (37–39 aa). Importantly, motif analysis of GLYIII proteins revealed proteins of cluster A and B differing with respect to two motifs, being exclusively present in the respective clusters. Indeed, proteins of cluster A had an extra stretch of amino acids, which were also evident from the 3D structures of these proteins and mainly represented loop regions in cluster-B proteins, but present as a beta-strand in cluster-A proteins. This region of variation may be responsible for the difference in substrate specificity of the enzymes.

Further, these plant GLYIII sequences were also very close in similarity to CvGLYIII1 and CrGLYIII1 proteins from green algae, sharing 70–75% sequence similarity to CrGLYIII1. In fact, first or N-terminal domains were more similar to CrGLYIII1 than the second or C-terminal domains. CrGLYIII1 shared 76% sequence similarity (at 91% query coverage; e-val: 5.61E-74) to DJ-1 family protein from *Chloroflexi* bacterium (HGV28139.1), whereas no ortholog or paralog could be obtained for CrGLYIII2, which showed the highest similarity to type 1 glutamine amidotransferase domain-containing protein from *Hymenobacter gelipurpurascens*, a flavobacterium composed of environmental bacteria (49.42% similar at query coverage of 83%, eval: 4.18E-34) and thus, resembled bacterial GLYIII more than the plant ones. Importantly, unlike *C. reinhardtii*, *C. variabilis* another green alga, has only one DJ-1 protein. In such case, it was possible that CrGLYIII2 had been acquired through horizontal gene transfer and hence, constitutes a case specific to *C. reinhardtii* as also, analysis of DJ-1 proteins in other green algal species revealed mostly single gene/protein being present in them [27].

Not only the cluster-A, B, and C proteins differed in their enzyme properties, differences in their subcellular localization patterns also existed. Most of the proteins of cluster-A were predicted to be cytoplasmic in nature with few exceptions mainly in monocots, being chloroplastic proteins and include CrGLYIII2 as well. Since DJ-1 proteins have a role in oxidative stress response, it is believed that their cytoplasmic localization may directly buffer cytosolic redox changes [21]. On similar lines, the human DJ-1 protein is known to be cytoplasmic under basal conditions, but its presence in mitochondria and nucleus is also realized [52] probably to allow the protein to shuttle between different functions based on the redox conditions of the cell. In the cytoplasm, human DJ-1 overexpression can protect against oxidative stress-induced cell death through the suppression of cytoplasmic TDP-43 aggregation [53]. Likewise, AtDJ-1a protein is also a cytosolic protein that facilitates protection against stress by activation of cytosolic SOD [22]. Similar to HsDJ-1 protein, Hsp3101 (SpGLYIII2) and SpDJ-1 (SpGLYIII1) from *S. pombe*, are dually localized proteins, being detected in both cytoplasm and nucleus, whereas SpGLYIII3 (or Hsp3102) is exclusively cytoplasmic [8]. Nuclear localization indicates the role of DJ-1 proteins in regulating gene expression as reported for HsDJ-1 protein, which regulates thioredoxin 1 expression via Nrf2-mediated transcriptional induction [54]. Importantly, cluster-C proteins were predicted to be multi-organelle localized, whereas most of the cluster-B proteins were cytoplasmic, with few being predicted in chloroplast and/or mitochondria as well. Mitochondrial localization of DJ-1 proteins is now well-established to be involved in cytoprotective effects on mitochondria. Human DJ-1 protein is in fact, known to translocate to mitochondria under oxidative stress to protect the cell from damage caused by ROS. To some extent, the translocation was shown to be facilitated by cys-106 oxidation in the human DJ-1, but may not be a prerequisite for translocation [52]. In mitochondria, human DJ-1 was detected both on the outer mitochondrial membrane by subcellular fractionation of M17 neuroblastoma cells [55] and also in the intermembrane space and mitochondrial matrix [56]. In *S. cerevisiae*, in the absence of DJ-1 paralogs, cells exhibited a significant delay in cell-cycle progression due to mitochondrial hyperfusion and partial DNA damage [57].

Further, chloroplastic localization of DJ-1 was observed in several plant DJ-1 proteins, from cluster B and C. AtDJ-1c (or AtGLYIII6) from cluster-C is a chloroplastic protein, which is essentially required for chloroplast development as mutants lack thylakoid membranes and granal stacks [23]. Another protein, AtDJ-1b (or AtGLYIII1) belonging to cluster-B, is also chloroplast-localized, which can act as an oxidation-robust holdase or a GLYIII enzyme. These discrete functions of AtDJ-1b respond differently to H_2_O_2_ with only GLYIII activity being sensitive to H_2_O_2_ [15].

## 5. Conclusions

The DJ-1_PfpI proteins have come a long way in the evolutionary journey. From modest origins in archaea, these proteins have acquired multiple functions to enable organisms to adapt better to their environment. The shift from aquatic to terrestrial habitation may have been the driving force leading to the evolution of special features of these proteins in plants. In fact, their sessile nature is a major contributor to the multigenic expansion of GLYIII family, which enabled them to supplement their defenses and other critical functions. In lower plants, these proteins acquired an additional DJ-1_PfpI domain, either by fusion or duplication, to help the plants better cope with the terrestrial conditions. Also, with the evolution within plants, the proteins started mobilizing to chloroplast as well. As the plants evolved further, these GLYIII proteins followed three distinct evolutionary paths to give rise to three categories of two-domain GLYIII proteins, each of which possesses specific motifs. These additional motifs may impart added physiological advantages to the GLYIII proteins. Future research is needed to dig out the specific functions of the diverse members of the GLYIII family, which may pave the way towards breeding for higher and sustainable stress tolerance in crop plants mediated by the GLYIII superfamily.

## Data Availability

All data and material were available in the supplementary files.

## Supplementary Materials

The following are available online at https://www.mdpi.com/2076-3921/10/5/648/s1, File S1: Sequences of GLYIII proteins used in the study; Figure S1: Multiple sequence alignment of domain sequences of GLYIII proteins from different plant species; Figure S2: Multiple sequence alignment of GLYIII proteins from different plant species; Table S1: Details of GLYIII proteins used in the study. Table S2: List of domains present in GLYIII proteins from different organisms; Table S3: Similarity scores of plant GLYIII proteins with those from *E. coli*; Table S4: Motif patterns observed in domain sequences of all GLYIII proteins.

## Abbreviations

GLY	glyoxalase
GSH	glutathione
MG	methylglyoxal
