# Supplementary material for: Tracing the Evolution of Plant Glyoxalase III Enzymes for Structural and Functional Divergence
Source: Antioxidants (Basel). 2021 Apr 23;10(5):648. doi: 10.3390/antiox10050648 (PMC8170915; doi:10.3390/antiox10050648)
Supplement: Supplementary file 1 [file antioxidants-10-00648-s001.zip › Kumar et al Supplementary files/Kumar et al Supplemental File S1.docx]

**Tracing the evolution of plant glyoxalase III enzymes for structural and functional divergence**

Brijesh Kumar^1#^, Charanpreet Kaur^1,2#^, Ashwani Pareek^2^, Sudhir K Sopory^1^, Sneh L. Singla-Pareek^1^*

1Plant Stress Biology, International Centre for Genetic Engineering and Biotechnology, Aruna Asaf Ali Marg, New Delhi-110067, India.

2Stress Physiology and Molecular Biology Laboratory, School of Life Sciences, Jawaharlal Nehru University, New Delhi-110067, India.

*Corresponding author: [sneh@icgeb.res.in](mailto:sneh@icgeb.res.in), ORCID: 0000-0002-0521-2622

#Authors with equal contribution

>CrGlyIII1

MASAGVAPLEKTVLVPIGNGTEEMEAVIIIDVLRRAGAKVTVASVEDSPQVVCSRSVRLVADTLIGDCASNTYDLIALPGGMPGAERLRDCGELEALVRAQRDSGRLYAAICATPYVFLQAKGLLDGKVATAHPAFSDGLKDQSRAMDRVVVDGNLTTSRGPGTAFEFALSLVKQMYGEDKAKMVGGPMVMFQYFV*

>CrGlyIII2

MLVPARRSAFTSTSGLVEFTHSYTVFKNAGCSVTVASPKGGLGPADSQGFAFYANDPDSKPLVAKRPDGSSYVPLTENTTAAGSITNAQLANFDILFFVGGTGSMWDFANDANLHRIIRVMWESGKVVSAVCHGPMALVHAKLSDNSSLVAGRAMTGFSNAEEEWLGSSNNVCGLCYPGNEATGCSAGLSPANCSGPHMPTEYAKQGSFLLEDGLKASGAIYMSTQQDWARFYFRPHVVR

HGRLVTGQNPGAGRETAEAAYDTHRLLRMTKKKNCPIWSQFADTFCSPVQQTAADPPVKCKTNGYIPKC*

>EcsGlyIII1

MIGREKYAGALGLLLGLTGFLCYLLLSPPTGSGRSLAWVPGGPAEAAMAAAAPAGAKHVLVPVADGSEEIESVTIIDTLVRAGALVTVASVGPEVEVTCSRGVKIKADCKIAECEARDWDAVVCPGGMPGAVSLKENETLEAILRKQNSEGRIVGAICAAPAVVLASHGLLEGKQATCYPASVFQSKIPQLVSEKVVVDQNLITSQGPATSMAFALQLVGSLFGQEKSDEVAKGLLFN*

>PhtGlyIII1

MPKVVILVTAAPDLNGHKTGLWLEECAAPYYIFKDKGYEVVLASPSAGPVPIDQVSVSDDFFTDDSKKFLHDAEAVGALSHSVELGSIDMSTVDCIYLAGGHGACVDFINNPSVTKAVQAIYAANKTVASVCHGVIALADCHQLDGTPLVKGRKVTGFSNSEEEAVELTEVVPYLLESKLKEQGCEYEKGENWSSKVCVDGNLITGQNPQSSAEVARTIVEILG*

>ThpGlyIII1

MSKRVLVPIADDSEEIETTCITDTLVRFGAEVVVASVKPGGELVCKMSRGVKMVADISIDEAVGQEWDLVALPGGMPGAEHLRDSATLISILEKQKASGKLYGAICASPAVVLATKGLIGEGATCFPADGLRSKMASPVDEDVVVQGNVVTSKGPGTALKFGVKLGELLYGEERAKEVAGGLLI*

>TgGlyIII1

MGEERPVCLGVLALQGAFFDHIKSFKRLNLGTRLRLTLVKKPADLEEIDALVIPGGESTAMRIIAGEEMMSALQAFVHEKKKPVWGTCAGCILLSNTVCTLETSSSPSASEIRETPSDDGYGDFIGGAAVRTCRNFFGRQVDSFEAPLRAVGRLKLAADGMHAICIRAPAIVDVSPEVEVLAYIDLPGRHTSVIAAAAHGPLLLTIFHPELTNDTRLHAFFLDNFVFPALGRASSAKATKREVEASGARNSEPSVSFPSGERRVSERAQAAQDAKSPQLGIRSS*

>NgGlyIII1

MTKQILLLAGDFVEDYEVMVPFQALQMIGYDVHTVCPEKEGGDSCPTAIHDFEGDQTYTEKPGHEFELNHDFDAVDPTEYDALVVPGGRAPEYLRTYDDVLEIVQHFFEAEKPVASLCHGLQILAAADVLEGRTCTAYPALEADVIAAGAEWEDDVTRDENLVTAQAWPDHPEWLAEFLEVLGTDIDHAEPAAADD*

>NgGlyIII2

MSETTAEIVLFDGFDELDAIGPYEVFENGAELGVALETRLVTLEGDTDEDGLVTASHGLCVEPERTLGEPDLLVVPGGGWTTEGGVRAVVEDGSIPAAVDECYTAGATVASVCTGGMILSEAGLLEGRPAATHHVAVDDLAETDANVVDERVVDDGDVLTAGGVTSGIDLALWLLEREFGEDVADAVSERMVHERRGEVFG*

>NgGlyIII3

MTKALFVVSEEGYWGAECIEPLETLSDAGVEVTVATPSGGPPEIDEQSIDPGEVGEETAEHIQTVHENDERLTDPIPTAQADADDYDAVVFPGGHGTEWDVNQDSDARRLLRDAVAGDDGTALVVCHAVGLLAFARDSHGAFVVNGRDVTGFPNAWETDIVDENDCMPSGRKLPYWVEDEVKVAGGNWDAELESETSVTVDGDLLTARGPESSTAAADRLLDELDELDG*

>VdGlyIII1

MAKAKILIIAGDAVEALELFYPYYRLKEEGWDVDVAAPSKKDLRTVVHDFEPGWETYSEKPGYLFKWVTKTLSEVRPEEYDGLVIPGGRMPEYVRVVALEDIKRIVRHFFETGKPVAAICHAPQILAAAGVIKGRRMTSYIAVRPEVENNGGIWVDQEVVVDGNLVTSRAWPDNPAWMREFIKLVKARIGSV*

>VdGlyIII2

MKALVIVADMSYEGEYSMAVQALRQLGMEVNSGLISKNANVNFDIDLTDKLGEDVLGGYDVVIFIGGYWAYYAVTGKEMPGRVKPMVNREAFEKLLTQSVSGGKKTILPLATPAYAAKLGLLRGKRATVYPTTDLIGILRGNGVDYVNEDFVIDGNVITLKRITVEFLTKALNK*

>MbMGlyIII1

MAIAYLMVFDGLSDWEPGLVVAEINKSNDYQVKTVGFNQDIVITMGGVSIVPDCTFKEIKYNDAAILILPGGKMWENDPVMDILPVVKKFIDLKIPVAAICGPTVFLAHHGFVENVKHTSNGREYLKSLIGEYEGSNLYVNQPSVSDKRIITANGIASVEFARDILDELDIYDKETLKSWYDFFKPPWLED*

>MbMGlyIII2

MKVYIYILNTLADWEIGYLTAELNSGRYLDKTRPPVELITIGNTTEPIKTMGGITITPDENIDNIKFEEDDLLILPGADTWTEEENKKIIDIVSRIIDEKVIIAAVCGATIALANKGILNNRKHTSNDIEVLKMFCPEYTGENFYLNQPAVTDDNLITASGIAPLEFSYEVLKRTNLMKTETLEAWYQLYKTKEPKYFHALMESIEGA*

>NoGlyIII1

MTNALFVVSEEGYWGEECVEPLETLSDAGVEITVATPSGGPPKLDETSADPDEVGEETAEHVREVHESDERLNDPIPVARADGETYDAVVFPGGHGTEWDVNQDSDARRLLREAVEGEDGKALVVCHAVGMLGFARDSQGAFIVNGREVTGFPNEWEEEIVDENDVMPDGRKLPYWVADEVEVAGGEFEPELDQDTSVTVDGDLLTARGPESSSAAADRLLEELDL*

>NoGlyIII2

MSEQILLLAGDFVEDYEVMVPYQALQAVGHDVHAVCPEKEAGDQCPTAIHDFEGDQTYTEKPGHNFELTHDFDAVEPSDYDALVVPGGRAPEYLRTYDEVLETVRHFFEDEKPVASLCHGLQILAAADVLEGRTCTAYPALEVDMRQAGAEWADDVVRDGNLVTAQAWPDHPEWLAAFLDLLGTDLEQPAEAAED*

>NoGlyIII3

MTADNSSDEREPETTGGEREPRTDGSGTDPGSTGTSDDAGGEIDENSKHEQLEEVRENSDGEHLTTDHGVKVSDTDNSLKAGERGPTIMEDFHFREKMTQFDHESIPERVVHARGTGAHGYFQPYEDPDLGEYDDISELTKASLFQDPDKKTPVFTRFSTVVGSRGSSDTVRDVRGFATKFYTEDGNWDLVGNNMPPFFIQDAMEFPDLVHAIKPEPDDGIPQASAAHDTFWDFASLKPEITHMIMWVLSGRALPRAYRMMQGFGVHTFRLVNDDGESVFVKFHWTPKLGTHQLVWDETTKLWGKSSDFNRKGLYDVIEEGYDPEWELGVQIFDEEQAAEFDFDVLDPTKIVPETEVPVRPIGKMVLNETPDNFFAEVEQVAFHPGNVVPGIDFSNDPLLQGRLFSYQDTQLNRFGGANWDEIPINRPIAERHNNQRAGFMRQEINEGKVSYKPNSIGDDDPQEAPAEEGGYEHYAEKVDGQKIRNRSDSFENHFTQARLFWNSMSEPEKQNIVDAAHFELGKVERVEIRERMVYDLFNNVDHEFAKRVAEGIGVEPPEEPGDEMPTHDREDPSLSMENRTPDTIETRKIAMLIDDGFDDEHVSKLRSALEEEGARVKIISKVLGEKSGVDGETVEPDKHHVAAASVSFDAVVIPGGDESVDAMRGQGDPKHFVAEAFKHYKPIAAVGEGTELFEAVDLPDTEIADEGDLVSDAGVVTCRSDDLESFAEAFVDAIAQHRHWDRDPEEVPA*

>NpGlyIII1

MTDALFVVSEEGYWGEECVEPLETLSDAGVEVTVATPSGSPPVIDERSLDPDEVGEETAEHVREVHETDERLNDPIPVAQADAEGYDAVVFPGGHGTAWDINQDKHARQLLRDAVEGNGKALVVCHAVGILGFARDSHGAFIVNGRDVTGFPNAWEEGIVDEQDRMPDGRKLPYWTEDEVKAAGGNWDAELDADTSVTVDGDLITARGPGSSSAAADTLLEELDVELEA*

>NpGlyIII2

MADIAAEIVLFDGFDELDAVGPYEVLRNGARAGASLETRLVSLAETDLVRASHDLRLEPDGTLGEPDLLLVPGGGWTTEGGVRAAVEDGALPEAVRERYDDGATIASVCTGAMVLSAAGILEGRPAATHPVAVDDLAATAATVVDERVVDDGDVVTAGGVTSGIDLALWLLEREFGVEIAEAVAAEMAHERRGDVFESS*

>NpGlyIII3

MTEHRILLLAGDFVEDYEVMVPFQALEMIGHEVHAVCPEKEAGDTCPTAIHDFEGDQTYTEKPGHQFELTHDFDAVEPSEYDALVVPGGRAPEYLRTYDEVLEITRHFFAEDKPVAALCHGLQILAAADVLEGRTCTGYPALEADVRGAGAEWTGEVTRDGNLVTGQAWPDHPEWLAEFLEVLGTDVDHAEPAAADD*

>MmGlyIII1

MEASIFLADGVEECEALLVVDLCRRAGIEIQTVAVNDRSGEAARTIHSSHKVSIICDTHLDDYQTAGEKILVVPGGLPGVNNLKANQKLSEILKAQGASAGRLAAVCAGPTVLGNLGLLDGKRATVFPGFDDGLGAAKYEDVPTVVDGQVITGRALGAGIEFALQIVTALRDKETAAKVAKQIVYPAIG*

>AgGlyIII1

MPTPPQLATDKKAAVLLAAGCEEVEALAVVDALFRAGIRADLVSVSQSLEVVSSHGVRIIADALIADVELADYDLLYLPGGMPGTLHLKACPAVPLEILRRADVGEPIAAICAAPSILAELGVLDRRRATANPAFMEAIAQGGATAEEKPVVVDGAITTSRGAGTAFDLGLELVRQMLGDGAADAVRAGIVHSR*

>ApGlyIII1

MTDDVKLLMLAGDYVEDYEIMVPYQALLMVGYKVDVICPGKKAGDKVLTAIHDFEGAQTYSEKPGHAFVLNADFDAIDTDEYLGLIIPGGRAPEYLRLNARVLEIVKAFADRPIAAICHGAQLLAAAGIIKGREVSAYPACRPDVELAGGTYADIPVTEAVTDGELVTAPAWPAHPAWLAQFLKVLGANVSI*

>RCGlyIII1

MSLLKKSQAATYGHDKGNDGMNVMSKSPAAKRSLVFFLVPHFTMLPFAAAVETLRIANRMLGYSAYTWRLCSADGEKVYSSSGIGIEVNSSLADERRNLSGENRPNMVLVCSGIYVEEFNNKSVNAWLRETYNRGIAVGSLCTGAHVLAQAGLLNGKRCAIHWENLPGFAEAFPQAEVYADLYEVDSNLYTCAGGTASLDMMLNLIGQDFGENLVNRVCEQQLTDRVRNPHDRQRLPLRARLGVQNAKVLSIIEVMENNLSEPLSLVEIADDAGLSRRQIERLFRQEMGRSPARYYLEIRLDRARHLLVQSSMPVVEVAVACGFVSASHFSKCYREVYNRSPQQERAERKLNMNTSRTGVVV*

>RCGlyIII2

MKLQVLALDPVFDTGLSTMLDVFSMANALSAMLSLEVPTFDVSVIGVRKKVKTAHGLAVPISGSFDPKAGGWLVVPAIACQMPDVLVPALDRPEIRDAGAVLREAKVAGMNIAAACVGTFVLAESGLLDGEKSTTSWWLAPLFRQRYPSVQLEDGRMLVKSGSLVTAGAALGHMDMALWLVRQVSPELAGLVARYLIVDNRPSQSAYVITDHLSHADPLVQRFERWARGRLSEGFSLDDAADVVGASKRTLARRMQQVLGKTPLSYFQDLRVERAVHLLKTSDKSPDEIAALVGYADGVTLRALLRQKTGRGVREVRAGG*

>HYGlyIII1

MAKVAVLLAPGFEEVEAIAPIDILRRGGVEVLIVGVKDKVIPSARNVKIEVDVTIDELKDVDNLDMIIIPGGMIGVENLKKSEEVKNLINQMNAKKKYVSAICAGPLVLKNAGVVENKHITSHPSVKLEFNEHLYKEESVVEDENIISSRGPATAMVFGFRLLEKLTSKEKAKEVAKAMLFDY*

>PdGlyIII1

MNLLLKNKTNMAKVYEFLANGFEEVEALAPVDILRRGGVEVKTVSITGSHLVESSHGVPVKADLLIEELSNADDADLLLIPGGMPGAKNLKDDSRVGKALLRQSDSGRRIGAICAGPMVLGALGLLKGRRATCYPGFDKFLTGAEYTNELCTVDGNITTGKGPAASFLYGLRLLEQLTSPEKADEIKKGMLINELAGLH*

>RsGlyIII1

MAKKILMLVGDYVEDYEVMVPFQALQMVGHTVHAVCPDKQAGESIATAVHDFEGAQTYSEKPGHRFTVNASFADVDPAAYDALVVPGGRAPEYLRLDPRVLDIVRHFSGAGKPIAAICHGAQLLSGAGVLKDRACSAYPACGPEVTAAGGTYQDIPVDQAHTDGNLVTAPAWPAHPAWLAQFLEVLGTRVLH*

>CaGlyIII1

MNALIILHPGFEEIEAITPIDLLSRADVHVTQVSIGPDKSVTGRSGITVSTSQLLGDCEDQDYDLVILPGGPGIQQIRHHPQLCRRLQQQAAADRWIACICAAPLLLKDAGLADGLQLCCHPSAEPDFTVTSSEAVVIDGKIITSRGAGTAHEFGLSLISALKGKALADQIAKSICWPH*

>VbGlyIII1

MPATTPTALVIVFDGIEEIEALTPVDILRRAEIKVTVASVNGLPTVTGRNQITFAADTSITRVAEDSFDLVILPGGPGVLELLENQAVSHILVAQDKAQKELAAICAAPKVLANHGILNSRKATSHSSVRDALPRPSDDPVVIDSHITTSQGLGTAVDFSLTLVKKLKGEALAQKIAESIHHSSRIF*

>PbGlyIII1

MSSVLVPLFEGFEEIEAVTIIDVLRRANIEVITASLDQLTVMGAHAIAIIADTTLDRVEPSKFDAIVLAGGAGTFRLREDPRIAAILKAQAKVNKLFAAICAAPTVLSDAGLLKGKRATSFPAVKEQLEVGEYLTDAVVVDGNAITSRGAGTAMVFALKLVEVLQDEAIANKLAQDMLV*

>SPGlyIII1

MKQALLPLMQGFEEIEAVTIIDILRRGDVQVVTAGLESVIVDGGHGITIMADSLLEHVINQEFDLIVLAGGAGTFRLQADPRIIPMLQKHAGLGKLTAAICAAPLVLSASGLLTEKRATSYPAVKDQLVVGEYLNDLVVVDGNIITSRGAGTATEFALQLLELLQGKAIAEEVAGKIVFIKKTA*

>CabGlyIII1

MSKALVVLAPGFEEIEAITIIDLLRRADIQVTVAGLQPNAITGSHHITVVPDTDIQHVKHETFDMLILPGGQPGTNNLKSSETLLGWIKERFIQGKKLAAICAAPTVFHAAGITKNLKITSYPSEKKVFTDSQYLEEAVVKDGAIITSRGVGTAIPFALRLIEELKDRQTAQQVAERILFEGRW*

>DaGlyIII1

MGKVIVVLADGFEEIEAVSVIDILRRADVEVCAAGVKDGNVKGAHGLIVKPDSTLEDIDEDDYDMIVLPGGAVGAENIGKSKDADDILRKFKKDDKYIAAICAAPKILADKGLLNGCMATSYPSFKDAVAKDSDYQEAIVVVDENIITSRGPATAAEFAFTLVELLVEEDTAEKLREGMLFTDDDCE*

>DalGlyIII1

MTAISIDNNDNIDIIVAMKLHLLVCDGVFDLGLAALTDTVGLANAMSGSLPQAPAPIEITLVGVRRRIRTAQGLTVPVVTARGVPEPDVVLVPAFGEKMPDTLSARLTRPDVPDAVAVLQQWSTAGAHLGAACSGSFLLAESGLLDGHRATTSWWLGPMFRQRYPNVTLDESRMIVNSTHFTTAGTALAHVDLALRIIRGRSPALAALVARYLLVEARSSQAEFVIPDHLAHADPMVERFECWARSRLAKGFSLAEAASAAGTSERTLARRLQSVLGKTPLSYFQDLRVEHAVHLLRTGNASVDQVAAQVGYSDGVTLRALLRRKLGRGVRELRRGG*

>CjGlyIII1

MSKKVLIPLAQGFEEAEFIGIADVLKRARELNPDLEVVIASLNSELLVKGANSINIKADCSIEDVDIENLDAIALAGGFEGMMNLKNSNVILNIIKQLHSKNKIVAAICASPIVLNEAGVLEGEFACYPSCEVGLNGNRVNKAVVVNKNVITSAGPATAILFGLELAKKLCGDEIYQKLYEGMLLPLTK*

>CjGlyIII2

MSKKVLIPLAQGFEEAEFIGIADVLKRARELNPDLEVVIASLNSELLVKGANDISIKADCSIEDVDIENLDAIALAGGFEGMMNLKNSNVILNIIKQLHSKNKIVAAICASPIVLNEAGVLEGEFACYPSCEVGLNGNRVNKAVVVNKNVITSAGPATAILFGLELAKKLCGDEIYQKLYEGMLLPLTK*

>HbGlyIII1

MKRVLVPLAQGFEEAEFIGIVDVLRRAGVEVLVAGLESSGNIKGANGIEIKAELALSSIDINSLDGIALAGGFEGMQNLSNNAKILEIIQRLHKDKKIVAAICASPIVLNKAGVLSSEFTCYPGCEEGINAKRLNEAVVIRDNVITSAGPATAILFGLAITKALMGEDVYKGLYEGLQVPLTRA*

>FsGlyIII1

MQILVLLADGFEETEFVVPVDLWRRAGFKVTVASVSGADVVDGLHGIKVQADVALSKLEPTDFDAVFLPGGGVGVQNLKASAAVENTVCSLNDDNKWVLAICAAPTVLSKARILVDRKATCYPGCETDLVCREFSEERVVVDGNIVTSRGPGTAEEFALKCIAVMGGAELSQKIQSQIVAR*

>GhGlyIII1

MTKKVALFVENGSEELELIAPLDILRRANIQVDLISANNEEYITSSHDVKIIVDKKINDIDNILDYDAIVIPGGMPGSTLLRDNDKIIKFYQEMYNAGKLVAAICAAPIVLSKAGILEDKEVTSYPGFDKEINCKTYDKEKAVIADKNVITAQGPAVAILFGYEIVNYLLQDDTAQNISDGMLVPVLKNNL*

>BcGlyIII1

MKKILLLLADGFEAVEASVFTDVLGWNKWEGDGSTEVVTVGLRNKLTCTWNFTVIPEKTVDDIQLDEFDALAIPGGFEEAGFYRDAYSREFLHVIQHFYAKQKPIASICVASLTLGKSGILVGKKATTYSHPTSKRKEQLKNFGAIVQNDLIVQDGNIITSSNPGTAFDVAFLLLEKLTSKKNAEHVKDLMGF*

>BcGlyIII2

MKKILLLLADGFEAVEASVFTDVLGWNKWEGDGSTEVITVGLRDKLTCTWNFTIIPEKTVDNIQLDEFDALAIPGGFEEAGFYRDAYSKEFLHVIQHFHVKQKPIASICVASLALGKSGILIGKKATTYSHPTSERKEQLKNFGAKVQNDLIVQDGNIITSSNPGTAFDVAFLLLEKLTSKQNAKHVKDLMGF*

>CsGlyIII1

MNKKIFVMLANGFELIEAMSPVDVLRRAGLNVVTVSTMENTLEVESAQKVKVVADINISDINVGEGVMVVIPGGFPGYVNLRSDSRVVEIVKEYLNSNDKFVGAICGGPTTLGINGLIGDYKFTCHTSVKEEMASEKYEHKDVVVDRNLITSPGAGKSVEFGLALASVFVDEATITKVKKGMELI*

>CsGlyIII2

MKKKVLFIIPPERFNEEELIKPKNILVNKGIDVVISSTVTGEITGDYEGTVISSAIFSNLNPSDFDIISVIGGSGTIDYLWENPSLINYLKEAYNKNIIISGICAGAVAIAETNLLTNRTATCYPIDIMINKLIKHNVNYLEKNVVVHSDIVTSNGPDGAEDFGLALLNLYL*

>LwGlyIII1

MKEKKVAVFLTNGFEEIEAITPIDLLQRAGINVDTVSITSDNLVESARKVKIMTDKVIGKIDFLEYDMLVMPGGPGTENYFKSQLFLDNVLKFSKNIENKKVAAICAAPTVLASLGILKGKNAVCFPACEDDLLKGKAILKKDRAVVDGNIITSRSAGTAMDFSLAIISELLGKEVAGKIAKEIVL*

>EcGlyIII1 (Hsp31)

MTVQTSKNPQVDIAEDNAFFPSEYSLSQYTSPVSDLDGVDYPKPYRGKHKILVIAADERYLPTDNGKLFSTGNHPIETLLPLYHLHAAGFEFEVATISGLMTKFEYWAMPHKDEKVMPFFEQHKSLFRNPKKLADVVASLNADSEYAAIFVPGGHGALIGLPESQDVAAALQWAIKNDRFVISLCHGPAAFLALRHGDNPLNGYSICAFPDAADKQTPEIGYMPGHLTWYFGEELKKMGMNIINDDITGRVHKDRKLLTGDSPFAANALGKLAAQEMLAAYAG*

>EcGlyIII2 (YajL)

MSASALVCLAPGSEETEAVTTIDLLVRGGIKVTTASVASDGNLAITCSRGVKLLADAPLVEVADGEYDVIVLPGGIKGAECFRDSTLLVETVKQFHRSGRIVAAICAAPATVLVPHDIFPIGNMTGFPTLKDKIPAEQWLDKRVVWDARVKLLTSQGPGTAIDFGLKIIDLLVGREKAHEVASQLVMAAGIYNYYE*

>EcGlyIII3 (YhbO)

MSKKIAVLITDEFEDSEFTSPADEFRKAGHEVITIEKQAGKTVKGKKGEASVTIDKSIDEVTPAEFDALLLPGGHSPDYLRGDNRFVTFTRDFVNSGKPVFAICHGPQLLISADVIRGRKLTAVKPIIIDVKNAGAEFYDQEVVVDKDQLVTSRTPDDLPAFNREALRLLGA*

>EcGlyIII4 (elbB)

MKKIGVILSGCGVYDGSEIHEAVLTLLAISRSGAQAVCFAPDKQQVDVINHLTGEAMTETRNVLIEAARITRGEIRPLAQADAAELDALIVPGGFGAAKNLSNFASLGSECTVDRELKALAQAMHQAGKPLGFMCIAPAMLPKIFDFPLRLTIGTDIDTAEVLEEMGAEHVPCPVDDIVVDEDNKIVTTPAYMLAQNIAEAASGIDKLVSRVLVLAE*

>PGGlyIII1

MSKQVSTPLRRVSILAIDRVFASTLMQAKDFFHLASLRYGKQLGHGLTPAFETRLVSPDGKPVNSFSDVMMPVDGGLENADVIILPAFWDDFETLCGRYPQILPWLREQHARGAVLCGEATGVFWLAEAGLLNGKEATTYWRFFNAFAERFPKVYLNQDKHLTDADNLYCAGGTTSACDLYIYLIERFCGANVAQAVARDILYEVQRSYSPGRIGFGGQKLHQDVIILQIQHWLEEHFADKFRFEDVAREHGMSIRNFMRRFQTATGDKPLHYLQRLRIETAKGLLSGSRKSIKTISYEVGYDDASFFARLFRQHTELSPNQYRQQFQQAA*

>PGGlyIII2

MGQTLIERRQFSGGMKGKNLRYLNEQSADSRLTRTGFLLLEHFSLPAFTQALDTIITANLLRAGLFSSRTFGLGEGEVISDLGLVIRPDARIGAASLGDLDLLVVCGGYRTELKASEELSNLLRAAAERGVSLAGLWNGAWFLGRAGLLDGYRCAIHPEHRPALTEIAKATQVSSEPYVIDRDRLTASSPSGAFHMALDWIKSLHGKALVEGIEDILAFEESRYRRIKPDENLCVSAPLREVVKLMDANLEEPLELEQLAVYAGRSRRQLERLFKEQLGTTPQRYYLELRITEARRLLQHTELSQMEVLVACGFVSPSHFSKCYSAFFGYRPSREKRLVK*

>PGGlyIII3

MTLQIGFLLFPQVQQLDLTGPYDVLASLPDVQVHLIWKDLVPVTASTGLLLKPTTTFDDCPNLDVICVPGGAGVGPLMEDEQTLGFIKRQAAQARYVTSVCTGSLVLGAAGLLQGKRATTHWAYHELLPKLGAIAVQDRVVRDGNLFTGGGITAGIDFALVLAAELVGAEAAQLIQLQLEYAPAPPFNSGSPDTAPSAIVDEARLRAAASLKLRTEITERAAAKLNLH*

>PGGlyIII4

MKTVAMVLFPDFLLLDMAGPMEVFSVANRYLKAEDHYQLITLGTERGMLRASNGVQVQTDSHIDDPAQAFDLLLVPGGPGAYNEKHPALLAWLQRSVGQAGCYGSICTGAFVLGHAGLLDGYRVTTHWNYTERLIKAFPEARVETDQIYTEDRNLITSGGVTAGIDLALAVVARDHGKRVAQDVAKVLLVVMKRQGGQAQFSPLMAAVAPQETAITRVQNHVLEHLDEAFTVERMAGLANMSTRHFARLFARDVNMTPMEFLQNARIDCARNLLETTELPLKTVAYKSGFGSVRHMRFLFAEKLGLTPAQYREQFS*

>PGGlyIII5

MNKNVAIVVFPGVQALDVSGPMDVFAEANRFLPPEDHYRLEVIGLERGAMACSNGLNLSAHRHFSEAPDAYDLLLVAGGPQLPFMDFGKPFDTWLREACARARRFGSICNGAFMLARAGLLEGRTVTTHWNDAEALAQLCPSTQVEADRLYVEDGALYTSAGVTAGIDLSLYLLARDHGAEVALSVAKRLVVFTQRSGGQSQFSPFLTPHAEPTSAVAMVQLYVLANLTGDLTIADLANAANMSARNFSRVFAREARVTPAEFVESARVDAARVLLESTTAPLKTVAYQCGFRDAQHMRSVFNRRLGVTPQQFRLNFALML*

>PGGlyIII6

MPDSPGLVAILAYDGLCTFEFGIAVEIFGLPRPEFDFPWYEHRIVAVDQGPMRAMGGIHVLADGGLELLAQARTIIIPGWRDRAAPVPPALIDALRQAHAQGARLLSICSGVFVLAASGLLDGHGATTHWRYTEELARRFPTIAVDPDVLYVDSGQLITSAGSAAGIDACLHLLARDFGTQVANSVARRLVMSPQRTGGQAQFIPMPVSATPRNDLSRVMQWARERLHQPLEVRDLASEAAMSERTFLRRFTEASGQSPKAWLQHERLARARELLESSVYNTEQIAQRCGYRSVESFRVAFRSVVGVPPSVYRERFGRGVKAIS*

>PGGlyIII7

MSSVSLQTLTWPKSEPRDVVFIAYPRMSLLDLSGAQTVFWAASKALAERGQHGYQLHTASLEGGLINTIEGLAVDTRALRQLDMASLDTLVVPGAPDICQTLDDHAELTTWLRHESQAARRVVSVCSGAFLLAQAGLLDGRRAATHWAMCDTLRERFAAVEVDADAIFIQQGAVWTSAGVTAGIDLALALVEADCGREVALQVARELVVYLKRPGGQAQYSTLLQSQMQGSPQFDSLHLWLMEHSSDPKLTVERLAEQAKMSLRNFTRVYKQSTGHTPSRAIELFRLEAARRLLESSAHNIDQVARLSGFGDEERLRACFQRHLSISPRDYRSRFSREG*

>PGGlyIII8

MTSFNSGAQPQNRAPQSIGFLLLDNFTLISLASAVEPLRMANQLSGRELYRWSTLTVDGGQVWASDGLQITPDASMHKAPPLDTVIVCGGIGIQRTVTREHVSWLQSQARQSKRLGAVCTGSWALACAGLLDGFDCSVHWECLAAMQEAFPRVAMSTRLFTLDRNRFTSSGGTAPLDMMLHLISRDHGRELSAAISEMFVYERIRNEQDHQRVPLKHMLGTNQPKLQEIVALMEANLEEPIDLDELAVYVAVSRRQLERLFQKYLHCSPSRYYLKLRLIRARQLLKQTPMSIIEVASVCGFVSTPHFSKCYREYFGIPPRDERVGSNTTQQVAMLPLPQAIVMSPLSGPMSALSQARNESTFASVRL*

>PGGlyIII9

MTFRALITLAEGIDDLQSVTLIDVLRRAGVEVVAASIEGRRMLTCARGTRLTADGMLIDVPAQTFDLIVLPGGVVGAQHLAAHQPLQQLLKDQASAGRLFAAIAESPAIALQAFGVLRQRRMTCLPSASHQLLGCTFVDQSVVVDGNGITAQGSGGALVFALTLVEQLGGKALRAKVAGEMLV*

>PGGlyIII10

MSQDFYFLLMPGFSAIGFISAIEPLRVANRFRGELYRWHVLSADGGAVLASNGMSVNADAALEPLKKGATLLVVAGFEPLKFATPTLEHWLRRLDNEGVTLGAIDTGSFVLAEAGLLDGHRLTLHWEAIDAFKESYPQLSVTQELFEIDRRRITSAGGTASIDLMLDLIAQVHGPQLAIQVSEQFVLGRIRPRKDHQRMEVATRYGISNKKLVQVIGEMEQHSEPPLTTLQLAESIKVTRRQLERLFRLHLNDTPSNFYLRLRLEKARQLLRQTDMSVLEVSIACGFESPSYFTRSYRARFARCPREDRRTAQA*

>PGGlyIII11

MPKTIHVLAFANVQILDVTGPLQVFASANDIARQRGLPVPYAPSVIAREGGAVMSSAGLAVLAEPLPRQASDTLIIAGGWGIYLAAEDVPLVDWVREHAAKCRRVASVCTGAFLLAASGWLDGRRVVTHWTRCEQLAQQHPKLQVEANPIFINDGPVWTSAGVTAGIDLALAMVEEDLGRDIALDVARHLVVFLKRPGGQSQFSVTLALQNQGNRFDELHAWIAENLTCDLGVPTLAEQAGMSERSFVRHYRADTGQTPARAIELIRVETARRLLSDTGLPVKRIAANCGFGSEETLRRSFLRAIGVTPQAYRERFSVSAGADPVMP*

>TpGlyIII1

MSVRVYLFVAHGFEEVETITPLDYLRRAGIALTLVGVGAEQVVSTRGLRVSCDCSLEALCASPGIADAACAADAVLLPGGLENCHTLAACAAVRDFVMRVHLRGGLVAALCAAPARVLSAWNLLGSRRYTCYPGMEPAVFSAHDDGVGKRTEEEKSRALRKPERARVVRDGNLLTACAAGAAEEFSFAVIEALCGVEVAQSVRAQVVAR*

>TpGlyIII2

MSVRVYLFVAHGFEEVETITPLDYLRRAGIALTLVGVGAEQVVSTRGLRVSCDCSLEALCASPGIADAACAADAVLLPGGLENCHTLAACAAVRDFVMRVHLRGGLVAALCAAPARVLSAWNLLGSRRYTCYPGMEPAVFSAHDDGVGKRTEEEESRALRKPERARVVRDGNLLTACAAGAAEEFSFAVIEALCGVEVAQSVRAQVVAR*

>SthGlyIII1

MARVAVCLADGFEDVEAVTPIDLLRRAGVEVVVAGVTGKEVTGSRGVRIVTDALLSDLSPEDFDGMVLPGGMPGSSNLAASDAVRAWLSHCMKAGKTIGAICAAPAVVLGKAGLLEGRRFTCYPGMEKEVEGGTWEPSPVVKDGNLITSRGVGTAGLFGLELVRAFAGEEAYQKVGKATLIL*

>SchGlyIII1

MKFALFLAPGFEEGEAIITTDVLRRGQIKVDLVSITDQLEVTSSHQVVIKADQLVTEVKYEDYDGFILPGGKIGVDNLAKNSQIKEWLLKANHDEKTIAAVCAAPQILGHLGILDNREATSYPGCTEGMENSIYNDEMAAITDGHIITGASVGSTMNFALAIADRVLGPEKVFALQNELVIRD*

>MsGlyIII1

MNLLVIVDNNFQDVELTTVVSILKRSQQFNKIAFYNPCSRKATGQFEIVKFNNLETSVNLKDYQAVFVPGGKACFSFKDNKEIIKKIRHFFDKDKWVFAICDAPNALRYNNLIKEQNYTSFPSAWSKELRSNSNYQNKGVVVFEKLITAKSSYYSQHLAFEIIRTLFGEKEYKLSKKLAKG*

>DdGlyIII1

MTKKILLLLCKGFEVMEFTPFVDVMGWAREDDNNEDKADIQVVTCGLYNKMVTSTFGVKVQVDVLLGEVVKSLDEFDALAIPGGFENYSFYEEAYSEDVSQLIRDFDSKGKHIASVCVAALALGKSGILKGRNATTYRNSLREHSVRQQQLRDFGANVIADQSIVIDKNVITSYNPQTAPYVAFELLSRLSDENKAKKVKTLMGF*

>DdGlyIII2

MAIQVAMIIFDGFEEIECVTTLDLLRRANIRVDLISIDNDKKSIKGSHYIELVTEYKFQDFIETLSNYNGIIIPGGPGIVQQLTNQKLIDAIKRFGLLYSNEINNNNNNNNNNNNNNNNNNNNNNNIENNNNRFLAAICAAPQIFGKCGLLKGRKVTHFPGCNQFMQDSIELLDQTIVVDGNIITASSAGVTIPFALKIVEFLKGIDASNLLYSQINPIQVKSS*

>TcGlyIII1

MTKKVLVPAADGTEEIELTCITDILRRAEIQVTVASVMESQNLILSRGLKITTDSLLKDESAAAYDGVFLPGGLPGADHLGKNAHLKKILEEMRSQGKWYGAICASPVSALAPMGMLEGVKTVTCYPAMKEKIPSHVHWSTDPVVRCGKCLTSKGPGTAIAFGLAIVAALLTKDCALRLAKELLVDETPFVEKALSNF*

>TcGlyIII2

MEVKTRWILRHYRMCVCVRLCFFFSIIYHHPPILLFQCVFICFFSFFLLLLLHGIPPQHMEWKIIILIIILQHYNIQKQQTKRKTMTKKVLVPAADGTEEIELTCITDILRRAEIQVTVASVMESQNLILSRGLKITTDSLLKDESAAAYDGVFLPGGLPGADHLGKNAHLKKILEEMRSQGKWYGAICASPVLALAPMGMLEGVKTVTCYPAMKEKIPSHVHWSTDPVVRYGKCLTSKGPGTAIAFGLAIVAALLTKDCALRLAKELLVDETPFVEKALSNF*

>TcGlyIII3

MTKKVLVPAADGTEEIELTCITDILRRAEIQVTVASVMESQNLILSRGLKITTDSLLKDESAAAYDGVFLPGGLPGADHLGKNAHLKQILEEMRSQGKWYGAICASPVSALAPMGMLEGVKTVTCYPAMKEKIPSHVHWSTDPVVRCGKCLTSKGPGTAIAFGLAIVAALLTKDRALRLAKELLVDETPFVEKALSNF*

>PfGlyIII1

MSGKKTALVAVASGSEDVEYITVVDVLRRAGVHVTTASVEKSEQVCLQSKNVVLADTTISKVRNNIYDVLVIPGGMKGSNTISECSEFIDMLKEQKANNRLYAAICAAPETVLDRHSLIDDVEAVAYPSFERNFKHIGKGRVCVSKNCITSVGPGSAVEFGLKIVEHLLGRQVALSLASGFLLHPAVTF*

>PteGlyIII1

MQNQNKQVLVPVGDGCEEIETVAIIDILRRANIDVTFASIKPVEDEKAPVIVGRSGISFICDTYLTEAVLKQQFDLIALPGGLSNAQSLGTHQPLLDRLRQQQEEGKWIAAICASPQLVLDKNGFMINSTGTCHPAHVQDYKGQFSEDRVHVSNKFITSRSPGTAIEFALALVELLVDQHTAVQMAKSLLVKR*

>EhGlyIII1

MKALVVIANGSEELEAVTIIDILARAKIQVTTATINSNLETACSRGVKIMADKFLSECNEQYDVIAIPGGLPGADNLAGSQLLIQKIKEQLAANRFVAAICASPAIVLEGNGIIEGRKCTAYPSFQPKLANQSAVHQRVVVDNHLITSQAPGSAIEFSLEIIRQLKGEEAMREVEKPLVLSFKY*

>LdGlyIII1

MNVLVVAADHSEDIELISIIDVLSRAGIKVTLASVMESKSITLAHGVNVMCDALIGEVSAVEYDAVLLPGGMPGAVHLGNNEALKKILQNARVGKKLYGGICAAPAVALAPMGLLEGVDTVTCYPGFEDKLPSSVKYSTNAVVKSENCLTSRGPGTAIYFALAVVSILKSPDLAERLAKAMLVDHSNEMNDVRAIK*

>SjGlyIII1

MVKVCLFVTGGSDEIETASCYGVFTRAKTPIDTVYVCEENKERLVNMLCGIRLYADRSLSEFQSAEDFMKEYDVVIIPGGWGGSLERSIPGTKMVQEIVRGMYKKPGKWVAMICAGSMGVMTSGLDPKTLELTSHACVIDVLRNAGYNWVDEPVVVSNNLITAQGPGTSMLFALKIAEQVLDKETYQQVYASLEMPQRN*

>SjGlyIII2

MVKVCLFVADGSDEIEFSAPYGIFTRANTPIDTVYVGDNKDRLVNMSRGIQLYAKRSLSEFQSTEEFVKEYDVAIIPGGWQGSLTLSGNKKVQEIVKEMYNKPGKWVAMICAGSLTAKTSGLGVKTLTSHPCITKDLQEAGYEWKNESVVVTDNLITSQGPGTAMLFALKIAEQVLDKDTYQKVYDSLEMP*

>AnGlyIII1

MTDSSTPSPLRIGVLLFPGFQALDVFGPLDVLNVLSWSPTTTPPVTLSLLSTTLSPISTLPPNFPHALSQSILPTATLSSSPPLDVLIIPGGWGTRAPLPEYTEYIRTVYPTLKYLLTVCTGGKLAARAGVLDGKRATTNKNDWEGVVRDAPGVQWVKEARWVVDRSDVGGDGKGTEVWSSAGVSAGVDLMFAWVESIWGEEVAAGVERVLEFRRWREGDVDPFVRD*

>CgGlyIII1

MSRNGDREGGKKPLNFGIVVFPAFQALDVFGPLDALNLLSRSYEMNLSVIAETLDPVSTKQIPGAQQQPGAIPATGPAVPAANSDFGQTILPTHTFQTAPPLDVLIVPGGQGTRYAGIRASIDFIKERFPQLQYLITVCTGAGVAARAGVLDGKRATTNKLSWEQTIALRPEVNWVHKARWVEDGNVWTSSGISAGIDVTFAWISAVYGKDLAKNIADRMEYTPVEDSSWDPFADRWGSKKA*

>CgGlyIII2

MKFTISIIAASLTAVVAGNPVQARVDTINGTNCTTALPVNYGALIFNGLDMIDIWGPLDVLQLNAHAYNMNVHLIAPTMDPIIAGVVNASDPTLNKFGSNFWPVIQPTATFADDLDLDVLIVPGGPGVRAAGLEPIVEYIKEMYPKVKYLITICTGASLAARAGVLDGKRATTNKRAWAQMTAFGPKVNWVAPARYVIDGNIWSSSGVTSSLDLTYAFVAEVYGQNQSTLIANTMEHTPLAADDDVFTDIWSVPHTNN*

>CgGlyIII3

MTLAQDESSVQACIPRPTTTTPASPPPTSLPRTFGMIISRAFEMLDVFGPLDALGMLARIHQLNLYLIAETMDPVTVEPVSAAMNAKNSSFFPKILPTHTFATAPTDIEVLMIPGGLWTRSPNLNSTIAYVRATYPKLRYLVSICTGASIAARAGVLDGRRATTNKASWASTIAYGPNVTWVPKARWVVDGNVWTSSGISAGIDATLAFIQDVYGRENATYIADLMEYEWHEDSGWDPYAEKFNVTGS*

>CgGlyIII4

MATKLGDKKSLRIGVMLEAVQLSDIMGIDLFGNISLEYYDKVKAFDPGFAPFEGLAPEIKFYFISSTLEPAEMTPGLKFVPNITYDDCPRDLDLVLIGGPLPSHRPPQADRFMKEAFVKTRVWMTTCIGSPWLASAGVLKGKKATVNREFLAFARQVHPDTEWLDQRWVVEEKEYDGEGKGELWTAGGAGAGLSMIIEYLNQNFDPAYVKKIALQGIGMEEIETNQFYKTIYGSGSVKP*

>CgGlyIII5

MSFDLSKPNRKIHAGVILTKGITEMLDVAPFEFFHCFQWSGDKETAAAMNLPENTMDDGLKFELHWVTEDGKPAKLASNAQILPTDSFESCPPLDIALMGAHDFKYKTSPAEIDFIRKTFEQCSAFLTICGGMVPLLEAGILAGKTSTCPRMMFDLMKKTVPVVNWVDKRWARDGKLWTSSTLLNGTDLIRAFATETWGERTGMIEFFLDAGHYPDRDVDFKDFKGQNYQMKQLPA*

>ScGlyIII1 (YDR533C-Hsp31)

MAPKKVLLALTSYNDVFYGDGAKTGVFVVEALHPFNTFRKEGFEVDFVSETGKFGWDEHSLAKDFLNGQDETDFKNKDSDFNKTLAKIKTPKEVNADDYQIFFASAGHGTLFDYPKAKDLQDIASEIYANGGVVAAVCHGPAIFDGLTDKKTGRPLIEGKSITGFTDVGETILGVDSILKAKNLATVEDVAKKYGAKYLAPVGPWDDYSITDGRLVTGVNPASAHSTAVRSIDALKN*

>FgGlyIII1

MSPPTKYAVALFPGFQALDVFGPLDVLNFTSKRQHMEVSLLHASLDPVSTFVEGGPACIGQSVVPTHTYETAPDDIEVLLVPGGFGARDPENVMRVRQFVKERYPKLKYLLTVCTGSAIVAQTGILDGREATSNKRSFDWVLTQGANVKWARNARWVVDGNIWTSSGISAGIDMTYAFIAEQYGQDIADDTAKGSEYVRNTDPNADPFAV*

>CvGlyIII1

MAGANVRVLVPIGTGSEEMEAVITIDVLRRAGAEVTVASVEDDLTVVCSRQVRLVADKSIKDCAGDWDLIALPGGMPGAERLRDSAALTELVAKQKAANKLHAAICATPAVAFEPQGVLAGKKATAHPAFSAKLTNQAAVEQRVVVDGKLVTSRGPGTAFEFALALVKMLYGEEKMREVAGPMVMADGYDKAL*

>GaGlyIII1

LLYGKEKADEVAGPLVMRSNHGAEYNFKELNPVKWTVASSPQILVPIANGTEEMEAVIIVDVLRRAKANVVVASVEGSLEISASRKVKLVADVLLDEALNNPYDIIVLPGGLGGAQAFANSDELVDSLKKQRESGRFYGAICASPALVLEHHGLLKGKKATAFPAMCSKLSDTSEVENRVIVDGNLVTSRGPGTTMEFALAITEKFLGREKADELAEAMLFVH*

>SmGlyIII1

MAVRALVTPGLVRAVAFSSGRGSLGLSADRPKRRRSCSSSCVKIIAMASPKKVLVPVADGTEEMEAVIVIDVLRRGGAHVTVASVGQEPKVTASRGVKLVADAIVSECGDEKYDLVVLPGGMPGAEHLRDSKALEDITRGQAQEQRAYAAICAAPAVALESWGLLNGLKATCYPSFVSKLSDPSSAESRVVKDGLVVTSRGPGTAMEFALTLVEQLYGKEKTQEVSKGLILLEGKPTKLEFNQTAWAAKVPSKPQVLVPIANGSEEMEAVIIIDVLRRAGMGVVVASVEETLQIVASRKVKIEADNLIGEVSSAHFDAIFLPGGMPGAEHLRDSKELQSILARQAKDSRVYGAICASPAVVLEANKILAGKKATAFPAFQSKLSDQSAVEARVVIDGLVATSQGPGTAMEFALAIVDKFSGKDSAVKTAEAMLFSY*

>EtGlyIII1

MASKRALVILAKGAEEMETVIPVDVMRRAGIKVTVAGLAGKDPVQCSRDVIICPDASLEDAKKEGPYDVVVLPGGNLGAQNLSESAAVKEILKEQEKRKGLIAAICAGPTALLAHEIGFGSKVTTHPLAKDKMMNGNHYSYSENRVEKDGLILTSRGPGTSFEFALAIVEALNGKEVADQVKAPLVLKD*

>EtGlyIII2

METVIPVDVMRRAGIKVTVAGLAGKDPVQCSRDVIICPDASLEDAKKESAAVKEILKEQEKRKGLIAAICAGPTALLAHEIGFGSKVTTHPLAKDKMMNGNHYSYSENRVEKDGLILTSRGPGTSFEFALAIVEALNGKEVADQVKAPLVLKD*

>TchGlyIII1

MRRAGIKVIVAHLAGKDPVQCSRDVVICPDTSLEDAKKEGPYDVVVLPGVVKEILEEQEKRKGLIATICAGPAGLLAHKIGFGSKATTHPLAKDKMMNGSHYSYSENRVEKNGLILTSHGPGTSFEFALALVEAMNGKEVANPGKAPLVLKD*

>TchGlyIII2

MVVLPRDNLGAQNLSESAAMKEILKEQEKRKGLIAAICAGPTALLAHEIDFGSKVKTHPLAKDKTMNGSHYSYSENRVEKDGLILTRHGPGTSFEFALAIVEALSGKEVAD*

>TchGlyIII3

MASKRALVILAKGAEEMETVIPVDVMRRAGIKVTVAGLAGKDPVQCSRDVVICPDASLEDAKKESAAVKEILKEQEKRKGLIAAICAVCTAHAHELPKYGVKVGLRNDAAACCTGLLLGRRLLNRFGVDKIYEGQVEVTRDEYNVESIDGQPGAFTCYLDAGLARTTTGNKVFGALKGAKQVSEYIKNSVPDVMEEMYKKAHTAIRENPVYEKKPKKEVKKKKWNCPKMSLAQKKDRIFLLTLFVGPTALLAHEIGFGSKVTTHPLAKDKMMNGSHYTYSENRVEKDGLILTSRGPGTSFEFALAIVEALSGKEVADQVKAPLVLKD*

>TchGlyIII4

MASKRALVILAKGAEEMETVIPVDIMRRAGIKVTVVGLAGKDPVQGSRDVVICPDASLKDAKREGPYDVVVLPGGNLGAQNLSESAAMKEILKEQEKQKGLIAAICAGSTALLAHEIGFGSKVTTHPLAKDKTMNGSHYTYPENRVEKDGLILTSRRPGISFEFALAIVEALSGKEVADQLHPFHVIRINKMLSCAGTDRLQTGMRGACGKPQGMVARVHIGQVIMSIRTKLQNKEHVIEALRRAKFKFPG*

>TchGlyIII5

MASKRALVILAKGAEEMETVIPVDIVRRAGIKVTVVGLAGKDPVQGSRDVVICPDASLKDAKREGPYDVVVLPGGNLGAQNLSESAAMKEILKEQEKQKGLIAAICAGPTTLLAHEIGFGSKVTTHPLAKDKMMNGSHYTYSENRVEKDALLNLRQPWGGYLDELFG*

>TchGlyIII6

MASKRALVILAKGAEETEMVIPVDVMRRAGIKVTVASLTGKDPVQCSRHIVICPDASLEDAKKEGPYDMVVLTGGYLGAHNLFNSAAMKEILKEQEKQKSLIAAIYAGPTALLTHEIGFGSKVTKHPLAKDKMMNGNQYSYSENHV*

>HsGlyIII1

MASKRALVILAKGAEEMETVIPVDVMRRAGIKVTVAGLAGKDPVQCSRDVVICPDASLEDAKKEGPYDVVVLPGGNLGAQNLSESAAVKEILKEQENRKGLIAAICAGPTALLAHEIGFGSKVTTHPLAKDKMMNGGHYTYSENRVEKDGLILTSRGPGTSFEFALAIVEALNGKEVAAQVKAPLVLKD*

>CeGlyIII1

MAAQKSALILLPPEDAEEIEVIVTGDVLVRGGLQVLYAGSSTEPVKCAKGARIVPDVALKDVKNKTFDIIIIPGGPGCSKLAECPVIGELLKTQVKSGGLIGAICAGPTVLLAHGIVAERVTCHYTVKDKMTEGGYKYLDDNVVISDRVITSKGPGTAFEFALKIVETLEGPEKTNSLLKPLCLAK*

>CeGlyIII2

MAQKSALIILAAEGAEEMEVIITGDVLARGEIRVVYAGLDGAEPVKCARGAHIVPDVKLEDVETEKFDIVILPGGQPGSNTLAESLLVRDVLKSQVESGGLIGAICAAPIALLSHGVKAELVTSHPSVKEKLEKGGYKYSEDRVVVSGKIITSRGPGTAFEFALKIVELLEGKDKATSLIAPMLLKL*

>XlGlyIII1

MASKRALVILAKGAEETETVIPADVMRRAGIKVTIAGLNGKDPVQCSRDVMLCPDTSLEEARTQGPYDVVVLPGGNLGAQNLSESPVVKEVLKEQEAKKGLIAAICAGPTALTVHGVGIGKSITTHPLAKDKIVNPDQYKYSEERVVKDENFITSRGPGTSFEFALEIVCTLLGKEVAEQVKTPLLLKH*

>XlGlyIII2

MAGKRALVILAKGAEEMETVIPTDVMRRAGIKVTVAGLSGKDPVQCSRDVMLCPDTSLEEARTQGPYDVVVLPGGNLGAQNLSESPVVKEVLKEQEAKKGLIAAICAGPTALTVHGVGIGKTITTHPLAKDKIVNPDQYKYSEERVVKDENFITSRGPGTSFEFALEIVCTLLGKEVAEQVKTPLVLKD*

>XlGlyIII3

MAGKRALVILAKGAEEMETVIPTDVMRRAGIKVTVAGLSGKDPVQCSRDVMLCPDTSLEEARTQGPYDVVVLPGGNLGAQNLSESPVVKEVLKEQEAKKGLIAAICAGPTALTVHGVGIGKTITTHPLAKDKIVNPDQYKYSEERVVKDENFITSRGPGTSFEFALEIVCTLLGKEVAEQVKTPLLLKD*

>DmGlyIII1

MVFFGFPQISRHFSKFTKMSKSALVILAPGAEEMEFIIAADVLRRAGIKVTVAGLNGGEAVKCSRDVQILPDTSLAQVASDKFDVVVLPGGLGGSNAMGESSLVGDLLRSQESGGGLIAAICAAPTVLAKHGVASGKSLTSYPSMKPQLVNNYSYVDDKTVVKDGNLITSRGPGTAYEFALKIAEELAGKEKVQEVAKGLLVAYN*

>HvGlyIII1

MSGKTALLLLAEGAEEMESVITIDVLRRAKIEVTVAGLDEHLVKCSRNVRIQPDDILENIKDKMYDAVIIPGGLGGAKKLSESTVVKNILEKHFKHEKLIAAICAGPTVLDAHNVGKGKKVTSYPSLKDKMKDYTYVAEKVVTDGNLVTSQGPGTSFNFSLEIVKILVGSDIAEEVSTGMLL*

>AsGlyIII1

MIGDSVFTCLFQVTVAGLLGAGPVKCARRTTITPDVALADVKDRKYDVVVLPGGQPGSNSFAASDEVGGVLKNQQEAGRIVAAICAAPIALKSHGIAPGTLVTSHPSVHQKLVDGGYKYSEDRVVAVGNVVTSRGPGTAFEFALKLVELLVGEEKVKEISAPMILKL*

>MmuGlyIII1

MASKRALVILAKGAEEMETVIPVDVMRRAGIKVTVAGLAGKDPVQCSRDVMICPDTSLEDAKTQGPYDVVVLPGGNLGAQNLSESPMVKEILKEQESRKGLIAAICAGPTALLAHEVGFGCKVTTHPLAKDKMMNGSHYSYSESRVEKDGLILTSRGPGTSFEFALAIVEALVGKDMANQVKAPLVLKD*

>AtGlyIII1

MASSSLCHRYFNKITVTPFFNTKKLHHYSPRRISLRVNRRSFSISATMSSSTKKVLIPVAHGTEPFEAVVMIDVLRRGGADVTVASVENQVGVDACHGIKMVADTLLSDITDSVFDLIMLPGGLPGGETLKNCKPLEKMVKKQDTDGRLNAAICCAPALAFGTWGLLEGKKATCYPVFMEKLAACATAVESRVEIDGKIVTSRGPGTTMEFSVTLVEQLLGKEKAVEVSGPLVMRPNPGDEYTITELNQVSWSFEGTPQILVPIADGSEEMEAVAIIDVLKRAKANVVVAALGNSLEVVASRKVKLVADVLLDEAEKNSYDLIVLPGGLGGAEAFASSEKLVNMLKKQAESNKPYGAICASPALVFEPHGLLKGKKATAFPAMCSKLTDQSHIEHRVLVDGNLITSRGPGTSLEFALAIVEKFYGREKGLQLSKATLV*

>AtGlyIII2

MASAVQKSALLLCGDYMEAYETIVPLYVLQSFGVSVHCVSPNRNAGDRCVMSAHDFLGLELTLNANFDDVTPENYDVIIIPGGRFTELLSADEKCVDLVARFAESKKLIFTSCHSQVMLMAAGILAGGVKCTAFESIKPLIELSGGEWWQQPGIQSMFEITDCVKDGNFMSTVGWPTLGHGIKLLLESLGGKVCSLEKKQASVLFLIGDYVEDYGINVPFRALQALGCKVDAVTPNKKKGEVCATAVYDLEDGRQIPAEKRGHNFFVTASWDDICVDDYDCVVVPGGRSPELLVMNEKAVALVKSFAEKDKVFAAIGQGKLLLAATGVLKGKRCASGKGMKVMVKVAGGEAVMEKGCVTDGKVVTAASATDLPAFLFDLSTALGLTVMF*

>AtGlyIII3

MANSRTVLILCGDYMEDYEVMVPFQALQAFGITVHTVCPGKKAGDSCPTAVHDFCGHQTYFESRGHNFTLNATFDEVDLSKYDGLVIPGGRAPEYLALTASVVELVKEFSRSGKPIASICHGQLILAAADTVNGRKCTAYATVGPSLVAAGAKWVEPITPDVCVVDGSLITAATYEGHPEFIQLFVKALGGKITGANKRILFLCGDYMEDYEVKVPFQSLQALGCQVDAVCPEKKAGDRCPTAIHDFEGDQTYSEKPGHTFALTTNFDDLVSSSYDALVIPGGRAPEYLALNEHVLNIVKEFMNSEKPVASICHGQQILAAAGVLKGRKCTAYPAVKLNVVLGGGTWLEPDPIDRCFTDGNLVTGAAWPGHPEFVSQLMALLGIQVSF*

>AtGlyIII4

MASFTKTVLIPIAHGTEPLEAVAMITVLRRGGADVTVASVETQVGVDACHGIKMVADTLLSDITDSVFDLIVLPGGLPGGETLKNCKSLENMVKKQDSDGRLNAAICCAPALALGTWGLLEGKKATGYPVFMEKLAATCATAVESRVQIDGRIVTSRGPGTTIEFSITLIEQLFGKEKADEVSSILLLRPNPGEEFTFTELNQTNWSFEDTPQILVPIAEESEEIEAIALVDILRRAKANVVIAAVGNSLEVEGSRKAKLVAEVLLDEVAEKSFDLIVLPGGLNGAQRFASCEKLVNMLRKQAEANKPYGGICASPAYVFEPNGLLKGKKATTHPVVSDKLSDKSHIEHRVVVDGNVITSRAPGTAMEFSLAIVEKFYGREKALQLGKATLV*

>AtGlyIII5

MGSMAQKSVLMLCGEFMEAYETIVPLYVLQAFGVSVHCVSPGRKTGDKCVMAAHDLLGLEIYTELVVDHLTLNANFDGVIPDQYDAIIIPGGRFTELLSADEKCVSLVARFAELKKLIFTSCHSQLFLAAAGLLTGGMKCTAFESMKPFIELSGGAWWQQPGVQTLFEITDCVKDGSFMSTMGWPTLGHSLKVLLESLGSKISSSKENHQTSLLFLIGDCVEDYSINVPFKAFQALGCKVDAVTPTKKRGEKCATIVHDLEDGRQLPTEKFGHNFYVTVAWDDVSVDDYDCIVVPGGRSPELLVMNPKAVELVRKFVEKGKFVAAIGMGNWLLAATGALKKKRCASSYGTKVAVKVAGGEIVESERCVTDDKLVTAASTSDLPAFLYALSTALGLSVVF*

>AtGlyIII6

MGSLGYSISMIASLSPTLMESRLISSMGCVSMTVAPSFSSVSVVSSSLGTTRRDRTLKLRSSMSPGMVTTLDSDVGVGSSATTKKVLVPIGYGTEEIEAVVLVDVLRRAGADVTVASVEQKLEVEGSSGTRLLADVLISKCADQVYDLVALPGGMPGAVRLRDCEILEKIMKRQAEDKRLYGAISMAPAITLLPWGLLTRKRTTGHPAFFGKLPTFWAVKTNIQISGELTTSRGPGTSFQFALSLAEQLFGETTAKSIEEFLLLRDGYQNPKNKEFNSIDWSLDHTPRVLIPVANGSEAVELVSIADVLRRAKVDVTVSSVERSLRITAFQGTKIITDKLIGEAAESSYDLIILPGGHTGSERLQKSKILKKLLREQHESGRIYGATNSSSTVLHKHGLLKEKRTTVYPSESDEPMNQQMIEGAEVVIDGNVITSLGLATVTKFSLAIVSKLFGHARARSVSEGLVHEYPRQ*

>OsGlyIII1

MAAQASPPTKKVLVPIVAGTEPVEAAVPIDVLRRAGADVTVASADDGELVVEVMYGVRIVADALVAGGDCAAAHFDLIVLPGGVPGAANLGGCAALEAMVRRHAATGGLYAAICAAPPLALASWGMLNGLKATAHPLFVDKFPPEVAAVDASVVVDASAVTSRGPATSTEFALALVEQLYSKNKAEQIAKEMLVRYDAGYTIDEVNSVQWKCNGTPKVLVPVANGTEEMELITIIDVLRRADADVVVASAENAGVEIVARHGMRIVADTTLDEAAADDQTSSFDLIILPGGTPGAKTMSSNEKLVTLLKKQAAASKPYGAIGAATAHVLEPHGLLEGKKAADQDGGDECESRVVVDGNVITSGGTGTAMEFAVAAVEKLLGRDVAQRVAEGLLFA*

>OsGlyIII2

MAMAAASASAMARRAASWPRLLLLSRAFAAAAAEPKRVLVPVADGTEPVEAAATADVLNRAGARVTVATADPAGDDRGLLVEAAFGVKLVADGRVADLEGEAFDLIALPGGMPGSANLRDCKVLEKMVKKQAEQGGLYAAICATPAVTLAHWGLLKGLKATCYPSFMEKFTAEIIPVNSRVVVDRNAVTSQGPATAIEYALALVEQLYGKEKSEEVAGPLYVRPQPGVDYVIDEFNSVEWKCSGTPQVLVPVANGSEEMEALNLIDILRRAGANVTVASVEDKLQVVTRRHKFNLIADIMVEEAAKREFDLIVMPGGLPGAQKLSSTKVLVDLLKKQAESNKPYGAICASPAYVLEPHGLLKGKKATSFPPMAHLLTDQSACDSRVVVDGNLITSKAPGSATEFALAIVEKLFGREKAVSIAKELIFM*

>OsGlyIII3

MAPKKVLLLCGDYMEDYEAMVPFQALQAYGVSVDAACPGKKAGDSCRTAVHQGIGHQTYAESRGHNFALNASFDEVNINEYDGLVIPGGRAPEYLAMDEKVLDLVRKFSDAKKPIASVCHGQLILAAAGVVQNRKCTAYPAVKPVLVAAGAKWEEADTMDKCTVDGNLVTAVAYDAHPEFISLFVKALGGSVTGSNKRILFLCGDYMEDYEVMVPFQSLQALGCHVDAVCPDKGAGEKCPTAIHDFEGDQTYSEKPGHDFALTASFDNVDASSYDALVIPGGRAPEYLALNDKVISLVKGFMDKAKPVASICHGQQILSAAGVLQGRKCTAYPAVKLNVVLGGATWLEPNPIDRCFTDGNLVTGAAWPGHPEFISQLMALLGIKVSF*

>OsGlyIII4

MLPSSRYLLAPAPLPAMVVRPPPPHPPSRGTSPLARPPLCRAMARAAPSLSAAASTAASSSTTPAKKKVLLPIAMGTEEMEAVILAGVLRRAGADVTLASVEDGLEVEASRGSHIVADKRIAACADQVFDLVALPGGMPGSVRLRDSVILQRITVRQAEEKRLYGAICAAPAVVLMPWGLHKRKKITCHPSFIEDLPTFRTVESNVQVSGELTTSRGPGTAFQFALSFVEQLFGPCKAEDMDNTLLTKVDDNLERSIEVNEIEWSSDHNPHVLIPIANGSEEMEIIMLTDVLRRANVNVVLASVEKSTSIVGSQRMRIVADKCISDASALEYDLIILPGGPAGAERLHKSSVLKKLLKEQKQTGRMYGGICSSPVILQKQGLLQDKTVTAHPSIVNQLTCEVIDRSKVVIDGNLITGMGLGTVIDFSLAIIKKFFGHGRAKGVANGMVFEYPKS*

>OsGlyIII5

MATRPLAASTLLPPLRFCSPLKTPPPSPPPPHLRRLQTLTRALASSSSAMASPPAKKVLVPIASGTEPMEAVITVDVLRRAGADVSVASVDPGSAQVGGAWGVKLAADALLDDLADAEFDLISLPGGMPGSSNLRDCKLLENMVKKHAGKGKLYAAICAAPAVALGSWGLLNGLKATCYPSFMDKLPSEVNAVESRVQIDGNCVTSRGPGTAMEYSVVLVEQLYGKEKADEVAGPMVMRPQHGVEFSLKELNSTSWNVGETPQILVPIANGTEEMEATMIIDILRRAKANVVVASLEETLEIVASRKVKMVADVLLDDALKQQYDLILLPGGLGGAQAYAKSDKLIGLIKKQAEANKLYGAICASPAIALEPHGLLKGKKATSFPGMWNKLSDQSECKNRVVVDGNLITSQGPGTSMEFSLAIVEKLFGRERAVELAKTMVFM*

>OsGlyIII6

MAPCKKVLMLCGDYMEDYEAAVPFYALAAFGVAVDCVAPGKKPPGDACLTAVHEFLGHDLYTELPGHRFAVTADFAAAAAADASRYDALVVPGGRFVERLSVDPLAVSLVAAFAGEGETATRRRPVVVTCHSQLLLAAAGAMRGVRCTAFFSMRRVVELAGGTWVEPDPLGLCVADGNVLSAIGWPAHGEIIRELLRAMGARVAGGRGQAVLFLCADYVDDYEANVPFRALAGVGCRVEAACPTKRKGEACVTAIYDATPAAASDERRGHNFAVTADWGDVDADRYACVVVPGGRAPELLATRGEAVALVREFAGKGKVVASIDQGHLLLAAVGLLDGRSCASGVATRVVAGLAGAASVRHGGAVADGKLVTAASWPDLAEFIAHIISLLGITVSF*

>BrGlyIII1

MIASLSPTLTEPMLISSMGSISAIVTSPSLYSISLIHSPIKQRKAQSLRLRASSASLSIDVDVVTIPKKVLVPIGYGTEEIEAVVLVDVLRRAGADVTLASVEQKLEVEGSSGTKLLADVLISKCSEQVFDLVALPGGMPGAVRLRDCGALEKIMKRQAEEKRLYGAISMAPAITLLPWGLLTRKKTTGHPAFFGKLPTFWAVQTNIQISGELTTSRGPGTSFQFALSLANQLFGETTAKSVGELLLLRDGFQNPEIKEVNSIDWSLNHTPRVLMPVANGSEEVEVVTISDVLRRAKVDVTVASVERSLRITASQGTKIVTDKLIGEAAESAYDLILLPGGRAGSERLQKSKFLKKLLKEQQEAGRIYGATNSASTVLHKHGLLKEKRTAVYVSDTDGPASDQMIEGAEVVIDGNVITSLGLATVTNFSLAIVSKLFGHGRARSVSEGLVHEYRGNLKAS*

>BrGlyIII2

MASSRKKVLIPIAHGTEPLEALAMITVLRRSGAYVTVASVENQVGVDACHGIKMVADTLLSDITNSTFDLIMLPGGLPGGETLKNCKPLENMVKKQETDGRLNAAICCAPALALGTWGLLEGKKATCYPVFMEKLAATCGTASESRVEIDGKIVTSRGPGTTIEFSLTLIEKLCGKQTAVDVSSILLPRPNPGEEFTFTELNQINWTFEDTPQILVPIAEGSDEIEAISVVDILRRAKANVVIASVSNSLEVVGSHKANLVADVLLDEVLEKSFDMIMLPGGLNGASRLSRSEKLVNMLKKQAEANKPYGGICASPAYVFEPHGLLKGKKATTHPVVSNRLSDQSHVDHRVVVDGNLITSRAPGTAMECALAIVEKFYGREKALQLAKATLV*

>BrGlyIII3

MAPSTKTVLIPIAHGTEPLEAVAMITTLRRGGADVTVASVETQVGVDACHGIKMVADTLLSDITDSIFDLIVLPGGLPGGETLKNCKPLENMVKKQDTDGRLNAAICCAPALALGTWGLLQGKKATGYPVFMEKLASTCATAVESRVQIDGRIVTSRGPGTTIEFSITLVEQLCGKEKADEVSSILLVRPNPGEEYTFTELNKTEWLFEDTPQILVPIAEDSEETEAIALVDILRRAKANVVIAAIGNSLEVVGSCEAKLVADVLLDEVAEKSFDLIVLPGGNGAQRFASCEKLVNMLNKQAEANKPYGGICKSPVYVFEHNGLLKDKKATTHPRVSNELSDQSHIDHRVVVDGNVITSRAPGTAMEFSLAIVEKFYGREKALQLAKATLV*

>BrGlyIII4

MGSSSVQKSALLLCGDYMEAYETLVPLYILQSFGVSVHCVSPNRNSGDRCVMAAHDFTGLELYTELVVDQLTLTASFDDVTPDNYDAIIIPGGRFTEILSADERCVDLVARFAELKKLIFTSCHSQVMLMAAGALAGGVKCTAFESMKTLIEFSGGEWWQQPGIQSMFEITDCVKDGNVVSTVGWPTLGHGVRVLLESLGGQVSSLKENQVSVLFLIGDYVEDYGINVPFRALQALGCKVDAVTPNKKKGEMCATLVYDLEEARQLPAEKRGHNFLVTACWDDVCVDDYDCVVVPGGRSPELLVMNPKAVALVKKFDEKDKVFAAIGQGKLLLAATGVLKGKRCASGKGMKVMVKVAGGEAVVSKGCVTDGKLVTAASASDLPAFLSGLSSALGVSVMF*

>BrGlyIII5

MASSTKTVMIPIAHGTEPLEAVAMITVLRRGGADVTVASVEDKVGVDACHNIKMVADTILSDITDSIFDLIVLPGGLPGGETLKNCKPLENMVKKQDTDGRLNAAICCAPALALGTWGLLEGKTATGYPVFMEKLAATCATASESRVEIDGRIVTSRGPGTTIEFSITLIEQLFGKDKADEVSSGLLVRPNPGEEFTFTELNQTNWSFQDTPQILVPIAEDSEEIEAIALVDILRRAKANVVIAAVGNSLEVVGSRKAKLVADVLLDEVAEKSFDLIVLPGGLNGAPRLASCEKLVNMLKKQAEANKPYGGICASPVYVFEPHGLLKGKKATTHPCVSNKLSDQSHIEHRVVVDGNVITSRAPGTAMEFSLAIVEKFYGREKALQLAKATLV*

>BrGlyIII6

MANSRTVLILCGDYMEDYEVMVPFQALQAFGVSVHTVCPGKKSGDSCPTAVHDFCGHQTYSESRGHNFTLNATFDEVDLTKYDGLVIPGGRAPEYLSMNASVVDLVKQFSSSGKPIASICHGQLILAAADTVNGRSCTAYATVGPALIAAGAKWVEPVTPDVCVVDATYEGHPEFIQLFVKAMGGKITGANKRILFLCGDYMEDYEVKVPFQSLQALGCQVDAVCPEKKAGDRCPTAIHDFEGDQTYSEKPGHTFGLTAAFDGVDSSSYDALVIPGGRAPEYLALNQHVLNVVKEFMNSGKPVASICHGQQILAAAGVLKGRKCTAYPAVKLNVVLGGGSWLEPDPIHRCFTDGNLVTGAAWPGHPEFVSQLMLLLGVQVSF*

>BrGlyIII7

MASPTKKVLIPVAHGTEPFEAVVMIDVLRRGGADVTVASVENQVGVDACHGIKMVADTLLSDVTDSVFDLIMLPGGLPGGETLKNCKPLENMVKKQNSDGRVNAAICCAPALALGTWGLLEGKKATCYPVFMEKLGATCATAVESRVEIDGRIVTSRGPGTTMEFSVALVEQLFGKEKAAEVSAPLVMRPNPGDEYTTTELNQIKWSYENTPQILVPIADGSEEMEAVAIIDVLRRAKANVVVAALGNSLEVVASRKVKLVADVLLDEAEKNSYDLIVLPGGLGGAEAFATSDKLVNMLKKQAESNKPYGAICASPALVLEPHGLLKGKKATAYPAMCNKLSDQSHIEHRVLVDGNLITSRGPGTSLEFALAIVEKFYGREKGLQLAKATLV*

>BrGlyIII8

MGSMAQKSVLMLCGEFMEAYETIVPLYFLQAFGVSVHCVSPGRKTGDKCVMAAHDLLGLEIYSELVVDHLTLNANFHEVIPEQYDAIIIPGGRFTELLSTDEKCVSMVARFAELGKIILTSCHSQLLLAAAGILGRGMKCTAFESMKPLIELSGGSWWQQPGVQTLFDITDCVMDGKFISTLGWPTLGNTLRILLESIGSKITCCKETQPSLLFLIGDCVEDYSINVPFKAFQALGCKVDAVSPNKKKGDKCATIVHDLEEGRQLPTEKFSHNFYVTVAWEDVSVDDYDCIVVPGGRSPELLVMNDKAVGLIKKFVEKGKFVAAIGMGNWLLAATGALKKKRCASGYGTKVAVKVAGGQILESEQCVTDDKLVTAATTSDLPAFVHALSTALGLSVVF*

>BrGlyIII9

MAFHCLNPITATPFSSTRLHRSTWRRTSRSFSVSATMASPAKRVLIPVAHGTEPFEAVAMIDVLRRGGADVTVASVENQVGVDACHGIKIVADALLSDVNDSVFDLIMLPGGLPGGETLKNCKPLENMVKKQDSEGRLNAAICCAPALALGTWGLLEGRRATCYPVFMEKLGATCGTAVESRVEVDGRIVTSRGPGTTMEFSVTLVEQLFGKEKAAEVSAPLVMRPNPGDEYTITELNQMNWSFDSTPQILVPIADGSEEMEAVAIIDFLRRAKANVVVAALGNSLEVVASRKVKLVADVLLDEAVKNSYDLIVLPGGLGGAEAFASSEKLVNMLKKQAESSKPYGAICASPALVFEPHGLLKGKKATAFPAMCNKLSDQSHIEHRVLVDGNLITSRGPGTSLEFALTIVEKFYGREKGLQLAKATLV*

>BrGlyIII10

MVSLGVSLSMIASLSPPSTMVAPSLYSASFISSPVLVPIGFGTEEIEVVVLVDVLRRAGAEVTLVSVEQKLEVEGSSGTKLLADVLISKCSEQVFDLVPLPGGMSGAVRLNCVTLEKIMKRQAEDKRLYRAISMAPAITLFPWGLLTRKKTTGHPAFFGKLPTFWAVKTTFRSQGSLQLAVDQALCSEEVEVVTVADVLRRAKVDVTLIGEAAESSYDLIILPKLLKEEQEAERIYGATNSSSTVLHKHGLLKEKRTVVYLSDTDMPVDDQMIQGAEVDGNVITGLGLATVTYFSLSIGGKLFGHGRPRSVSQGLVHEYQWNLKAS*

>PpGlyIII1

MAKCKAVLMIIGDYVEDYEVMVPFQALLAYGLKVDAVCPGKKAGETCATAIHDFLGHQTYSESKGHNFALTANFEDVDADSYDALVVPGGRAPEYLSLDENVLNLVKKFESAHKPIASICHGQLILAAAGVLKDKQCTAYPAVKPVVVAAGGIWKDPSPISACFTDGKLVTGAAWPGHPEFLKQTLAALSATVQGGDKKVLMLCGDYMEDYEAMVPFQAMQALGYQVDAVCPDKKSGDTCATAVHDFEGAQTYSEKPGHNFALTATFSEVKVQDYDALVVPGGRAPEYLSLNEKVLDLVREFDDAKKPIASICHGQQILAAAGVLKGKKCTAYPAVKSHVVLSGGQWLEPEPISKCFTDGHLVTGAAWPAHPEFVAQLMALLGTAVTF*

>PpGlyIII2

MEGQVGAGKRILILATSHDKLGNTNQATGCWAEELTAPYYIFKDAGAHVDVASIKGGKIPMDEASFSEGFVTDHVKRYLEDEELKQRVEHSLSVKDVSGNYDALFVPGGHGIIYDGPVDQDFIALGNRFWAEGKIISSVCHGPAGLVGMTAPDGTSIFKGKKVCGFSNAEEEAVGKTNVVPFLLEDKLKELGGLYEAGPNWHPKAAADGQLVTGQNPGSSAKVAELVLEALSK*

>PpGlyIII3

MEPMRVTRMLQVAIVIFPNITVLDFIGPYEPLNRLPNVNVVLVSHSKGIIRAEKSLIGFEATATFDEVQEPDIVIVPGGYGVNALIRDKPILDWIRKTHEKTLYTTSVCTGSLLLAAAGILNGLEATCHWRVLPELSKFGAKPTSSRIVESGKIITAAGVSAGIDMGLKLVALLSNDTTCKLIQLVIEYDPQPPFDCGSPAAAGPELVSMARAYAEKNYPDYFIF*

>PpGlyIII4

MAAMAISVLPIVSGLLPCLVLRVIEGQSRRDICNLSSALCPVSESLQFERSQAWCRTKVKESSRRSRRVDARVVMAQANSAMEKSPAKKQVLVPVANGTEEMEAVIVIDVLRRAGAAVTVASVEEGKLVNASRGVNLLADCLISECEGVEYDLVVLPGGMPGAERFRDSQVLKRITVKQSQEKRMFAAICAAPVVALQSWGLLAGLNATCHPGFAVKLEDKSSVGGRVVRDGALTTSRAPGTAFEFALALIEQLYGPESVPAVADPMVLPSHDGIVSAALKFNDEDWTTSSIPRVLVPVANGSEEMEVVIIVDILRRAGAEVVVASVESETTIKASRNVQLVADTLVSEIVQTKFDLVVLPGGMPGATRLQESEELSKILNQQVESGRCYGAICAAPAVVLEANGLLNGKKATSHPAFSSILKDQSAVEGRVVIDGRLITSRGPGTAMEFTLNIVEKLFSRSKAQEVAEPMVFNYV*

>PpGlyIII5

MDIHTMATKINPATELAPAENKKKVLVPVANGTEEMEAVIVINVLRRAGATVTVASVEEGMQIAASRGVNIVADCLISECEEQEYDLVALPGGMPGAERLRDSKALKSIAEKQVKAKRMIAAICAAPVVALQAWGLLKGLHATCHPSFTGKLEDKAAVESRIVRDSILTTSRAPGTAFEFALALVEQLYGPENLPTVSGPMVLPPDDGTDARALKFNDQEWSTSSTPQVLVPIVNGSEETEVVIIVDILRRAGAKVVVASVESEATIKAARNVQLVADTLISEVANTKFDLVVLPGGMPGADRLQKSKELMRILQEQAEEGRIYGAICAAPAVILESSGLLHGYCGELRSSGI*

>PpGlyIII6

MMLEKAKLQKSGGKNMKLVIRADAPLMLQGLIEPLKRLGIWSDQKHAEGIAAGWRLQEQAQKWKAECPDAAWDEAQWAAEEAALLKIMAHCPPDRQTASWFLQRLLVPADMPLSVLELLTGWEIREKAQWRSDSISLHAILWLKEIGAKPMPQLYGTLLRIERGDDLAVLVMDEETTGETEIPTLWEHRDHSVEHVDEGMLIMQVNLDDSSPEWLAYVLEQCMRAGANDVHFLPVTMKKSRPGTLLQVMCYQSAAEAIKTILFSETTTFGIRTFPVACHRLARRFATAQTQWGEVQVKLGYHRGKRVQVAPEYAVCAQLAEAAQVPLKQMSKKVLIVTGDAVEALEVFYPYYRCLEEGFETVIAAPNVKTLHTVLHDFEAHSETYTEKPAYQLAAHASFAEINPEAFDALIIPGGRAPEHIRLNEHLKPIVAHFFEANKPVAAICHGAQVLTIVREYIAGKEMTAYQACRPDVEACGAIYQTETLHVDGNLVSGHAWPDLPGFMREFLKLLR*

>PpGlyIII7

MRYLPALSVNDIVVDEYAALVIPGGSPHPLLQNQELIKVIRAFYDQEKLLAAICGGPSLLGAAGILQEIAYTASLEPTDLEYRDVMNWENKRSELLVIDKNVITATGSNYLHFAEEVLRKLGAVLPTEENPLQYFREPSLS*

>PtGlyIII1

MANCKPRKKVLLLCGDYMEDYEAMVPFQALQAYGIAVDAVCPGKKAGDYCRTTVGDSGAYHGYQTYTEKPGHNFSLNATFDEVDFSKYDALVIPGGRAPEYLAMNESVLNCRKCTAYRALGPVLIDAGALWIEPKTMMDCVSDGNLITGVIYKAHPEYIQLVVKALGGKIAGSDKRILFLCGDFMEDYEVTVPLQSLQALGCHVDAVCPKKKAGDFCPTAVHDFEGDQTYTEKPGHNFILTASYEGLDASSYDALVIPGGRSPEYLALDETVIALVKKFMQSKKPVASICHGQQILAAAGVLKVVSNVFFHFHRITQVIIFFTLAILILVYVNHVAAGKKVHRIPCCEAERRLGRGNMARTGSNRSLLHRRKPGYRSCMARAPSVCVSADGLTWYPSVILAVLLQ*

>PtGlyIII2

MANCKPQKKVLLLCGDYMEDYEAMVPFQALQAYGIAVDAVCPGKKAGDYCRTTVEDSGAYHGYQTYTEKPGHNFSLNATFDEVDFSKYDALVIPGGRAPEYLAINESVLNCARQFSDSGKLIAAICHGPLILAAAGLLKGRKCTAYHALGPVLIDAGALWIEPKTMMDCVSDGNLITGVIYKAHPEYIQLVVKALGGKIAGSDKRILFLCGDFMEDYEVTVPLQSLQALGCHVDAVCPKKKAGDFCPTAVHDFEGDQTYTEKPGHNFILTASYEGLDASSYDALVIPGGRSPEYLALDETVIALVKKFMQSKKPVASICHGQQILAAAGVLKGRKCTAYPAVKLNVVLGGATWLEPDPIDRCYTDENLVTGAAWPGHPQFVSQLMALLGIRVSF*

>PtGlyIII3

MANCKPQKKVLLLCGDYMEDYEAMVPFQSLQAYGIAVDAVCPGKKAGDYCRTTVGDSGAYHGYQTYTEKPGHNFSLNATFDEVDFSKYDALVIPGGRAPEYLAINESVLNCARQFSDSGKLIAAICHGPLILAAAGLLKGRKCTAYHALRPVLIDAGAHWIEPKTMMDCVSDGNLITGVIYKAHPEYIQLVVKALGDKIAGSDKRILFLCGDFMEDYEVTVPLQSLQALGCHVDAVCPKKKAGDFCPTAVHDFEGDQTYTEKPGHNFILTASYEGLDASSYDALVIPGGRSPEYLALDETVIALVKKFMQSKKPVASICHGQQILAAAGVLKGRKCTAYPAVKLNVVLGGATWLEPDPIDRCYTDENLVTGAAWPGHPQFVSQLMALLGIRVSF*

>PtGlyIII4

MESMLCLLSPSPTKLSPFKKLTSTCALKTTFSSLSFASMTSPPQPKTPSTKKLSSSKPTKTLSPKTPTTSTSVQETSTPFSPPLKKVLVPIGFGTEEMEAVIIVDVLRRAGAEVIVASVEPQLEVEAAGGTRLVADTSISKCANEVFDLVALPGGMPGSARLRDCEVLRQITSKQAEDKRLYGAICAAPAITLLPWGLLRRKQMTGHPAFMDKLPTFWAVASKIQVSGELTTSRGPGTSFEFALSLVDQLFGESVAKEVGQLLLMQADDDTQRKEEYNKVEWSFDHNPRVLLPIANGSEEIEIVAIVDILRRAKVDVVVASIEKSVQILASRGIKIVADKLIGDAAESVYDLIILPGGNAGAERLHKSKVLKKLLQEQYTAGRIYGAVCSSPAVLHRQGLLKDKRATAHPSVVTNLNNVSNGAKVVIDGKLITSKGLSTVTDFALAIVSKLFGHARTRCVAEGLVFDYPRS*

>PtGlyIII5

MSILLRHVIPPPHSVLYSTSEKSKLNFPSFRNPRFSFSSIKATAATPMASTTKKVLVPIANGTEPIEAVITIDVLRRGGADVTVASIEKQIRVDATYDVKLVADSLLSECSDAVFDLITLPGGIPGATNFKNCQLLEKLVKKQVEDGKLYAAVCASPAVAFGSWGLLNGLKATCHPFFMDELKSSGAITVESRVHEEGNVVTSRGPGTTMEFAVALVEKLFGKEKADEVSGPLVMRSNHGDEYSIKELNPMQWTFDNVPQVLVPIANGTEEMEAIIIIDILRRAKVNVVVASVEDSLEILASRKVKLEADMLLDEAAKLSYDLIVLPGGLGGAQAFAKSEKLVNMLKKQRESNRPYGAMCASPALVLEPHGLLKGKKATAFPAM*

>PtGlyIII6

MANCKPQKKVLLLCGDFMEDYEAMVPFQALEAYGIAVDAVCPGKKAGDCCRTVIQDSGAYHGYQTFTEKLGHNFSLNANFDEVDFSKYDGLLLPGGRAPEYLAINESVLDCVRKFSDSGKPIGSICHGHLILAAAGSVKGRKCTALHALGPVLIDAGAHWIEPKTRMDCVADGNIITGVIYRAHPEYIRLFVRALGGKVTGSDKRILFLCGDFMEDYEVTVPFQSLQALGCHVDAVSPKKKAGDICPTAVHDFEGDQTYSEKPGHNFILTASYEGLDASTYDALVIPGGRAPEYLALDETVIALVKEFMQSRKPVASICHGQQILAAAGVLKGRKCTAYPTVKLNVVLGGATWLEPDPIDRCYTDENLVTGAAWPGHPEFVSQLMALLGIQVSF*

>StGlyIII1

MASATKKVLVPIAIGTEPIEAIVPIDILRRAGAEVIVASVENQLQIEVMYGIKIVADALISDCVDTEFDLISLPGGVPGAANLGNCKILESIVKKQAENGKLYAAICAAPAVALGSWGLLKGLKATCYPSYMEELSSHAIAVESRVQKDAKVVTSRGPATSIEYAVALVEELYGKEKANEVSGPLVMRPNHSEEFAFADLNSVNWTLTSKPRILVPIANGSEEMEATIIIDVLRRANAQVVVASLEDKLEIVASRKVKLVADVLLDEAAKQSYDLIVLPGGLGGAETFAKSEKLVDMLKKQRESSKPYGAMCASPALVLEPHGLLKGKKATAFPALCNKLSDPSEAENRVVVDGNLVTSRGPGTTMEFALAIADKFIGHKETLELAKEMIF*

>StGlyIII2

MANQKRVLLLCGDYVEDYEVMVPFQALLAYGVAVDAVCPGKKSGDICRTAVHQLSGHQTYSESRGHNFALNATFDEIEASKYDGLFIPGGRAPEYLAMNESVLDLVKSFANAKKPIVAICHGQLILAAADVVSGRRCTAYPAVKPVLVAAGAHWEEPETLASCTIDGNLITGSTYEGNPEFIRLFVKALGGSIVGSGKRILFLCGDFMEDYEVKVPFQSLQALECHVDAVCPKKKAGEKCPTAVHDFEGDQTYSEKPGHDFTLNANFESVDVSSYDGLVIPGGRAPEYLALDDDVIKLVQEFMESKKPVASICHGQQILSAAGVLKGKKCTAYPAVKLNVILGGATWLEPEPIDRCFTDGNLVTGAAWPGHPEFISQFMALLGVHVKF*

>StGlyIII3

MANQKRVLLLCGDYVEDYEVMVPFQALLAYGVAVDAVCPGKKSGDICRTAVHQLSGHQTYSESRGHNFALNATFDEIEASKYDGLFIPGGRAPEYLAMNESVLDLVKSFANAKKPIVAICHGQLILAAADVVSGRRCTAYPAVKPVLVAAGAHWEEPETLASCTIDGNLITGSTYEGNPEFIRLFVKALGGSIVGSGKRILFLCGDFMEDYEVKVPFQSLQALECHVDAVCPKKKAGEKCPTAVHDFEGDQTYSEKPGHDFTLNANFESVDVSSYDGLVIPGGRAPEYLALDDDVIKLVQEFMESKKPVASICHGQQILSAAGVLKVSISVNII*

>StGlyIII4

MKSISPLFSVSTAKSPIFYFSPISQRLSSVKFAAPPKINSPDTKRPSSKSVKTLSAAPTIDPITTTAASASPKKVLVPIGFGTEEMEAVILADVLRRAGAEVTVASVEQQLEVEAYGGTRLVADTFISTCSTEIFDLVALPGGMPGSARLRDCEVLQKITSRQAEEKRLYGAICAAPAVTLLPWGLLKRKQTTCHPAFIDKISSFRVVKTNTRVSGELTTSRGPGTSFEFAICLVEQLFGEPVAREIGELLLMNPAGDDPKRQEFNEVGWSLDRTPQVLIPIANGSEEIEVVTLIDILRRAKVNVVVASVEKSAQVLASKGTKIVADKLINATSDSIFDLIILPGGAAGAERLHKSKILKKLLKEQESAGRIFGAICSSPAVLQKQGLIKDKKATAHPAVLDKLKDGVNDAQQKVLSISTPRARNASPETLRVPKYVKGATQIDGLHTYFTGNYKKGNFLVLDDLVADITSSRGDFPKISPNLQYRVPTRSLTGIREFTITRPGRIGNRDDQSNDASYGQIVLRLISVSSGHYIAPPAHKFLFFV*

>StGlyIII5

MKSISPLFSVSTAKSPIFYFSPISQRLSSVKFAAPPKINSPDTKRPSSKSVKTLSAAPTIDPITTTAASASPKKVLVPIGFGTEEMEAVILADVLRRAGAEVTVASVEQQLEVEAYGGTRLVADTFISTCSTEIFDLVALPGGMPGSARLRDCEVLQKITSRQAEEKRLYGAICAAPAVTLLPWGLLKRKQTTCHPAFIDKISSFRVVKTNTRVSGELTTSRGPGTSFEFAICLVEQLFGEPVAREIGELLLMNPAGDDPKRQEFNEVGWSLDRTPQVLIPIANGSEEIEVVTLIDILRRAKVNVVVASVEKSAQVLASKGTKIVADKLINATSDSIFDLIILPGGAAGAERLHKSKILKKLLKEQESAGRIFGAICSSPAVLQKQGLIKDKKATAHPAVLDKLKDGVNDAQVVIDGKLITSQGLATAIQFALAIVSKLFGHARARSVAEGLVYQYPKS*

>StGlyIII6

MPGSARLRDCEVLQKITSRQAEEKRLYGAICAAPAVTLLPWGLLKRKQVTIAFSL*

>ZmGlyIII1

MAAAAASSVARRAASRGGQFIARAFASGVGGGGEKAKRVLVPVAAGTEPIEAATTADVLNRAGARVTVATADPPGDDGLLVQAAYGVKLVADGRVADLQGEPFDLIALPGGMPGSVGLRDCKALEKMVKTHAEDGGLYGAICAAPAVTLAYWGMLKGLKATCYPSFMEKFTAEVIPVDSRVVVDRNAITSQGPGTAVEFALALVEKLYGKEKMEEVAGPLYLRPQHGVEYTIEELNSVEWKCGSTPQVLVPVANGSEEIEAVNLIDVLRRAGANVIVASVEEKLQIVTRRHKFNLIADMMLDEAIKMQFDLIVMPGGLQGAQKFASTKQLVDLLKKQAESNKPYGAICASPAHVLEPHGLLKGKKATAFPPMSHLLTDQSACEHRVVVDGNLITSRAPGTATEFGLAIVEKLFGRDKAVSIAKELIFM*

>ZmGlyIII2

MATRSLTSALPLSRTPRVPSHRPSLRRLQTLTRALTSSSPQAMASSPTPKKVLVPIANGTEPMEAVITIDVLRRAGADVAVASVEPGATSVAASWGIKLTADALLADLADDEFDLISLPGGMPGSSTFGECKVLEKMVKKHVEKGKLYAAICAAPAMTLGTWGLLNGLKATCYPSFIDKLPSEVHAVESRVQIDGKCMTSRGPGTAMEYSVILVEQLYGKEKAKEVAGPMVMRPQHGVEFSMKEVNSTSWNVGETPNILVPIANGTEEMEATMILDILRRAKANVVVASLEDKLEIVASRKVKMIADVLLDDALKEQYDLILLPGGLGGAEAYAKSDKLMDLIKKQAAANRLYGAICASPAIALEPHGLLKGKKVTSYPAMWNKLADQSECNNRVIVDGNLITSQGPGTSMEFSLAIVEKLFGRERALELAKSMVFI*

>ZmGlyIII3

MAAKKVLMLCGDYMEDYEVMVPFQALQAYGVSVDAVCPSKKAGDICRTAVHQLTGHQTYSETKGHNFTLNASFDEITASEYDGLVIPGGRAPEYLAMDEKVLDLVRMFSGAKKPIASVCHGQLILAAARVVENRTCTAFPAVKPVLVAAGAKWEEPDTMAKCTVDGNLITAATYNSHPEFISLFVKALGGSVAGSDKRILFLCGDYMEDYEVMVPFQALQALGCHVDAVCPDKGAGETCPTAIHDFEGDQTYSEKPGHDFTLTASFGSVDASSYDALVVPGGRAPEYLALNDKVISLVKAFAESGKPIASICHGQQILSAAGVLKGKKCTAYPAVKLNVLLGGGTWLEPDPIHRCFTDGNLVTGAAWPGHPEFVSQLMALLGVKVSF*

>ZmGlyIII4

MASPAAVKKVLVPIAAGSEPAEAFVPIAVLRRAGADVTVAAAGAGTGLRVHAMYGVTVVADASVADCADASYDLVVLPGGVPGADNLGGCAALEGIVRRHALGGGLCAAICAAPPLALARWGLLDGVKATAHPEFVDKFPAEVAGVDANVVVDGRVVTGRGPAAAMEFALALVDQLYGKGKVDEIAKPMMVRYEPGYAFEELNPVQWRCSGTPRVLIPVANGSEEMEVLVTVDVLRRAKADVVVASAEEVVVARHGTRIVADALLQDAAGQQFDLIVVPGGMPGVKTTLADKVELMALLKEHAAAGRAYGAIGAATAQVLEPHGLIGGSMKATTCASRADRPSECGSRVVVDGNLATSGSTGTAMEFALAVVEKLLGPEAAREVAEALLFV*

>ZmGlyIII5

MAAAAASSVARRAASRGGQFIARAFASGVGGGGEKAKRVLVPVAAGTEPIEAATTADVLNRAGARVTVATADPPGDDGLLVQAAYGVKLVADGRVADLQGEPFDLIALPGGMPGSVGLRDCKALEKMVKTHAEDGGLYGAICAAPAVTLAYWGMLKGLKATCYPSFMEKFTAEVIPVDSRVVVDRNAITSQGPGTAVEFALALVEKLYGKEKMEEVAGPLYLRPQHGVEYTIEELNSVEWKCGSTPQVLVPVANGSEEIEAVNLIDVLRRAGANVIVASVEEKLQIVTRRHKFNLIADMMLDEAIKMQFDLIVMPGGLQGAQKFASTKQLVDLLKKQAESNKPYGAICASPAHVLEPHGLLKGKKATAFPPMSHLLTDQSACEHRVVVDGNLITSRAPGTATEFGLAIVEKLFGRDKAVSIAKELIFM*

>ZmGlyIII6

MLSPWHPLLSPVAPPAMELRSPLNSHNCSLTSSIRPPPPRSLARAPPTLSATAAAVSSLSATSAAISSPSCPKKKVLVPIAMGTEEMEAVIIAGVLRRASADVTLASVEDGLEVEASCGSRIIADTHIASCADQVFDLVALPGGMPGSVRLRDSDILQRITVRQAEEKRLYGAICAAPAVVLVPWGLHRRKKITCHPSFIGDLPAFRAVESNVQVSGELTTSRGPGTTFQFALSFVEQLFGLRAAEDMDKILMAQTHDGLERSAEVNELEWSCGRNPDVLIPIANGCEELEIIILVDILRRAKINVVLASVEKYPAVLGSQRMKIVADKSIMSASDSIYDLIILPGGPAGAEQLHRSRILKKLLKQQMQAGRMYGGVCSALKVLQQEGLLEDKTVTAHHAVASELTCQVIDQPNVVIDGNLITGKGLGTVVDFALAIIRKFFGHGRAKAVANGIVFEYPKS*

>ZmGlyIII7

MASPAAVKKVLVPIAAGSEPAEAFVPIAVLRRAGADVTVAAAGAGTGLRVHAMYGVTVVADASVADCADASYDLVVLPATAHPEFVDKFPAEVAGVDANVVVDGRVVTGRGPAAAMEFALALVDQLYGKGKVDEIAKPMMVRYEPGYAFEELNPVQWRCSGTPRVLIPVANGSEEMEVLVTVDVLRRAKADVVVASAEEVVVARHGTRIVADALLQDAAGQQFDLIVVPGGMPGVKTTLADKVELMALLKEHAAAGRAYGAIGAATAQVLEPHGLIGGSMKATTCASRADRPSECGSRVVVDGNLATSGSTGTAMEFALAVVEKLLGPEAAREVAEALLFV*

>TaGlyIII1

MKGSANLGDCKQLEKMVRKHTQSGRLCAAIGAAPAMVLARWGVLKGFTATCHPALLGRLGDDDGVIAVDDRVVKDRNVVTSQGVGTAIEFALELVEQLYGELKAHEVAGPLYMRPQQGVKYSIQEYNQIQWKCTGTPRVLVPVANGSEEMEALNLIDVLRRAGARVTVASVEDTPRIVTRHYKLNLITDVMLEQAAEMEFDLIVMPGGLPGALKFTSSEKLVGMLKKQAESGRPYGAICASPAYVLEPHGLLKGKKATSFPPMAHLLTDQSACEYRVVVDGNLITSRAPGTATEFALAIVEKLFGEEKAVALAKELVFM*

>TaGlyIII2

MAAKRVLLLCGDYMEDYEAMVPFQALQAYGVSVDAVCPGKKAGDACPTAVHKPIGHQTYAESKGHNFALNASFDEVDAAAYDGLVIPGGRAPEYLAMDEKVLALVRKFSDAKKAIASVCHGQLILAAAGVVRDRTCTAYPAVKPVLVAAGAKWVEADTMKKCVVDGNLVTAAAYDGHPEFISLFVKALGGSVAGADKRILFLCGDYMEDYEVMVPFQALQALGCHVDAVCPDKGAGDKCPTAIHDFEGDQTYSEKPGHDFALNASFDSVDASSYDALVIPGGRAPEYLALNEKVLSLAKGFMDKGKPVASICHGQQILAAAGVLEGRKCTAYPAVKLNVVLGGGTWLEPDPIHRCFTDGNLVTGAAWPAHPEFVAQLMALLGIKVSFT*

>TaGlyIII3

MALSWHIAPAARSVPSKGEASRVHSVQACWLAWTLELGEIRSVSRARWGVSAAMAARRVLLLCGDYMEDYEAMVPFQALQAYGVSVHAACPGKKAGDACPTAVHKPVGHQTYAESKGHNFALNASFDEVDAAGYDGLVIPGGRAPEYLAMDEKVLDLVRKFSDAKKPIASVCHGQLILAAAGVVRDRTCTAYPAVKPVLVAAGAKWVEADTMKKCVVDGNLVTAAAYDGHPEFISLFVKALGGSVAGSDKKILFLCGDYMEDYEVMVPFQALQALGCHVDAVCPDKGPGDKCPTAIHDFEGDQTYSEKPGHDFPLNASFDGVDASSYDALVIPGGRAPEYLALNEKVLSLAKGFMDKGKPVASICHGQQILAAAGVLQGRKCTAYPAVKLNVVLGGGTWLEPDPIHRCFTDGNLVTGAAWPAHPEFVAQLMALLGIKVSFA*

>TaGlyIII4

LLKGKKVTAFPPMAHLLTDQSLCENRVVIDGNLITSRAPGTATEFALAIVEKLFGREKAVSIAKEFVFM*

>TaGlyIII5

MAAKRVLLLCGDYMEDYEAMVPFQSLQAYGVTVDAVCPGKKAGDACPTAVHKPIGHQTYAESKGHNFALNASFDEIDAAGYDGLVIPGGRAPEYLAMDEKVLALVRKFSDAKKPIASVCHGQLILAAAGVVRDRTCTAYPAVRPVLVAAGAKWVEADTMKKCVVDGNLVTAAAYDGHPEFISLFVKALGGSVAGADKRILFLCGDYMEDYEVMVPFQALQALGCHVDAVCPDKGPGDKCPTAIHDFEGDQTYSEKPGHDFALNASFDGVDASSYDALVIPGGRAPEYLALNEKVLSLAKGFMDKGKPVASICHGQQILAAAGVLQGRKCTAYPAVKLNVVLGGGTWLEPDPIHRCFTDGNLVTGAAWPAHPEFVAQLMALLGIKVSFA*

>TaGlyIII6

MMLDEAAKMEFDLIVMPGGLSGAQKFASTDKLVDLLKKQAGSGKAYGAICASPAHVLEPHGLLKGKKATAFPPMAHLLTDRSLCENRVVIDGNLITSRAPGTATEFALAIVEKLFGREKAVSVAKEFVFM*

>TaGlyIII7

MPGSVNLRECKVLERMVKMHAEKGGLYGAICAAPAVTLAHWGMLKGLKATCYPSFMEKFTAEVIPVNSRVVVDRNVVTSQGPGTAIEFALALVEQLYDKEKMEEVAGPLYVHPQHGADYTIEELNSVEWKCSGTPQVMIVH*

>TaGlyIII8

MLVRYECGYSMKEVNSVEWHCSGTPKVLLPLANGIEEMEAIILVDALRRAKADVVVASIEGGVEITARYGTRIVADVMLGEAADRAPFDLIIVPGGMPGAKTLGGCEQLVALLKKQAEANRPYGAIGAATAHVLEPHGLLKGRKATTCASMAGLLTDGSECENRVVVDGNVITSRSPGTAMEYAVAVVEKMLGRDEARRLAEGLLFLG*

>TaGlyIII9

MLDEAADRAPFDLIIVPGGMPGAKTLGGCEQLVALLKKQAEANRPYGAIGAATAHVLEPHGLLKGKKATTCASMAGLLAD*

>TaGlyIII10

MMLNEAAKMEFDLIVMPGGLSGAQKFANTDKLVDLLKKQAGSGKPYGAICASPAHVLEPHGLLKGKKVTAFPPMAHLLTDQSLCENRVVIDGNLITSRAPGTATEFALAIVEKLFGREKAVSIAKEFVFM*

>TaGlyIII11

MPGSTNLRECKVLERMVKMHAEKGELYGAICAAPAVTLAHWGMLKGLKATCYPSFMEKFTAEVIPVNSRVVVDRNVVTSQGPGTAIEFALALVEQLYDKEKMEEVAGPLYVRPQHGAEYTIEELNSVEWKCSGTPQVLVPVANGSEEIEALNLIDVLR*

>TaGlyIII12

MMLDEAAKMEFDLIVMPGGLSGAQKFASTDKLVDLLKKQAGSGKPYGAICASPAHVLEPHGLLKGKKATAFPPMAHLLTDQSLCENRVVIDGNLITSRAPGTATEFALAIVEKLFGREKAVSIAKEFVFM*

>TaGlyIII13

MPGSTNLRECKVLEKMVKMHAEKGELYGAICAAPAVTLAHWGMLKGLKATCYPSFMEKFTAEVIPVNSRVVVDRNVVTSQGPGTAIEFALALVEQLYDKEKMEEVAGPL*

>TaGlyIII14

KSRRSPPGRRAACLSGVSFALRPCPSGEPRAAASSPPLPSPSWLLPLLICRLLPRPADRRGGAPVTAFVQPDLDPDVWRSASAKVSWRARPRGRCGLYRFTCALRQKGCIFIGWILLVSELQIPMLSASKPLLSPSSLIAMPIRPPPHAPTHYAPPHRRTLPLRSIARAAPSSSTAATAVSLPPSPKKVLVPIAMGTEEMEAVILAGVLRRAGADVTLASVEDGLEIEASYGTRIIADKPIAACADQGGMPGSVRLRDNEILQRIMVRQAEEKRLYGAICAAPAVVLMPWGLHKGRKITCHPSFIGDLPTFRAVESNVQVSGELTTSRGPGTAFQFALSFVEQLFGPHAVEDVDSTLIDAALERSTEVNRVEWPFDHKPQVLIPIANGSEEMEIIMLVDILRRANINVVLASVDESTNVVGSQRMKIVADKCILGASDSKHDLIIIPGGPAGAERLHRSTTLKKLLKEQKQAGRMYGGISYSPLILQKQGLLEDKTVTAHPSIVDQLTCQVIDGSKVVIDGNLITGKGLGTVMDFSLAIVRKFFGHGRAKGVANGMVFDYPKSRNA*

>TaGlyIII15

MRPQHGAEFSMKELNSTSWNVGENPQILVPIANGTEEMEAIMIIDILRRAKANVVVASLESTLEIVASRNVKMVADVQLDDALKQQYDLILLPGGLGGAQAYAKSDKLIGLIKKQAEANKLYGAICASPAIALEPHGLLKGKKATSYPAMWSKLVDQSECKNRVVVDGNLITSQGPGTSMEFSLAIVEKLFGRERAFELAKAMVFV*

>TaGlyIII16

MPGSVRLRDNEILKRIMVRQAEEKRLYGAICAAPAVVLMPWGLHKGRKITCHPSFIGDLPTFRAVESNVQVSGELTTSRGPGTAFQFALSFVEQLFGPHAVEDVHSTLIDAGLERSTEVNRVEWPFDHKPQVLIPIANGSEEMEIIMLVDILRRANINVVLASVNESTNIVGSQRIKIVADKCILDASDSKYDLIIIPGGPAGAERLHRCTTLKKLLKEQRQASRMYGGICYSPLILQKQGLLQDKTVTAHPSIVNQLTCQVIDRSKVVIDGNLITGKGLGTVIFPGHCKKILWPWASERCGKWNGF*

>TaGlyIII17

MLSVSKPILAPTSLTAMAIRLPPHAPTHYALPHRRTPPFRSIARAAPSPSTTSTAVSLPPSPKKVLVPIAMGTEEMEAVILAGVLRRAGADVTLASVEDGLEVEASYGTRIIADKSIAACADQEFDLVALPVTHLSYYYYYFYFRYVYKRSKSFLFSLLKNDKTGDKQFCWHQHRCAGILKFWPD*

>TaGlyIII18

MPGSVRLRDNEILQRIMVRQAEEKRLYGAICAAPAVVLMPWGLHKGRKITCHPSFIGDLPTFRAVESNVQVSGELTTSRGPGTAFQFALSFVEQLFGPHAVEDVDSTLV*

>TaGlyIII19

MLLYESGGMPGASTFGDCKILENIVKKHAEKGKLYAAVCAAPAVALGAWGLLNGLKATCHPSVMDKLPSEVQAVESRVQIDGNCVTSRGPGTTMEYSVVLVEQLYGKEKADEVAGPMVMRPQHGAEFSMKELNSTSWNAGENPQILVPIANGTEEMEAVMIIDILRRAKANVVVASLEGTLEIVASRNVKMVANVLLDDALKQQYDLILLPGGLGGAQAYAKSDKLIGFIKKQAEANKLYGAICASPAIALEPHGLLKGKKATSYPAMWSKLTDQSECKNRVVVDGNLITSQGPGTSMEFSLAIVEKLFGRERALELAKSMVFV*

>TaGlyIII20

MPGSVRLRDNEILQRIMVRQAEEKRLYRAICAAPAVVLMPWGLHKGRKITCHPSFIGDLPTFRAVESNVQVSGELTTSRGPGTAFQFALSFVEQLFGPHAVEDVESTLIDAGLERSTEVNRVEWPFDHKPQVLIPIANGSEEMEIIMLVDILRRANINVVLASVDESTNIVGSQRMKIVSDKCILGASDSKYDLIIIPGGPEGAELLHRSTALKKLLKEQKQASRMYGGICYSPLILQKQGLLQ*

>TaGlyIII21

MAPSKKVLMLCGDYMEDYEAAVPFYALAGLGVAVHCAAPGKAPGDPCLTAVHDFLGYELYTELPGHRFRVTADF*

>TaGlyIII22

MLSTSKPLLAPTSLTVMAIRLPPHAPTHYAPPHWQTPPFRSITRVAPSPSTTATAVSLPPSPKKVLVPIAMGTEEMEAVILAGVLRRAGADVTLASVEDGLEVEASYGTRIIADKSIAACAHQVFDLVALPEGMPGSVRLRDNKILQRIMVRQAEEKRLYGAICAAPAVVLMPWGLHKGRKITCHPSFIGDLPTFRAVESNVQVSGELTTSRGPGTVFQFALSFVEQLFGPHSVEDVDSTLISQIDAGLERSTEVNRVEWPFDHKPQVLIPIANGSEEMEIIMLVDILRRANINVVLASVDKSTNIVGSQRMKIVADKCILGASDSKYDLIIIPGGPGGAERLHRSTTLKKLLKEQKQASRMYGGICYSPLILQKQGLLQDKTVTAHPSIVNQLTCQVIDRSKVVIDGNLITGKGLGTVMDFSLAIVRKFFGHGRAKGVANGMVFDYPKS*

>TaGlyIII23

ALVGKFAAEGKVVASIDQGHLVLAASGLLKGKRCASGVPMRVISNLAGAAAVVPEGAVADGKLVTAASWPDLAEFIAHLVDLLGITVSF*

>TaGlyIII24

MATRPPVFSTLTASFSPSPPHLLRRLQTLTRALASSSPQPMASSPPLKKVLVPIANGTEPMEAVITIDVLRRAGADVAVASVEPGAAQVAASWGVKLAADTLLADLAEADFDLISLPGGMPGASTFRDCKILENIVKKHAEKGKLYAAVCAAPAVALGAWGLLNGLKATCHPSVMDKLPSEVQAVESRVQIDGNCVTSRGPGTTMEYSVVLVEQLYGKEKADEVAGPMVMRPQHGAEFSMKELNSTSWNVGENPQILVPIANGTEEMEAVMIIDILRRAKANVVVASLEGTLEIVASRNVKMVADVLLDDALKQQYDLILLPGGLGGAQAYAKSDKLIGLIKKQAEASKLYGAICASPAIALEPHGLLKGKKATSYPAMWSKLADQSECENRVVVDGNMITSQGPGTSMEFSLAIVEKLFGRERAFELAKSMVFV*

>TaGlyIII25

MGTEEMEAVILAGVLRRAGADVTLASVEDGLEIEASYGTRIIADKPIAACADQVFDLVAVPGGMPGSVRLRDSEILQRIMVRQAEEKRLYGAICAAPAVVLMPWGLHKGRKITCHPSFVGDLPTFRAVESNVQVSGELTTSRGPGTAFQFALSFVEQLFGLHAVEDVDSTLIDAALERSTEVNRVEWPFDHKPQVLIPIANGSEEMEIIMLVDILRRANINVVLASVDESTNVVGSQRMKIVADKCILGASDSKYDLIIIPGGPAGAERLHRSTTLKKLLKEQKQAGRMYGGISYSPLILQKQGLLEDKTVTAHPSIVSQLTCQVIDSSKVVIDGNLITGKGLGTVMDFSLAIVRKFFGHGRAKGVANGMVFDYPKSRNA*

>SpGlyIII1

MVKVCLFVADGTDEIEFSAPWGIFKRAEIPIDSVYVGENKDRLVKMSRDVEMYANRSYKEIPSADDFAKQYDIAIIPGGGLGAKTLSTTPFVQQVVKEFYKKPNKWIGMICAGTLTAKTSGLPNKQITGHPSVRGQLEEGGYKYLDQPVVLEENLITSQGPGTAMLFGLKLLEQVASKDKYNAVYKSLSMP*

>SpGLYIII2

MASEGKVLLVASSYYGPFYPDGMNTGVHFAELLIPYQVFREAGYEVQLTSETGKCKFDDHSIKKSALGEVERDAFDNKDNEFWYALKDIKPADKINYKEFCIMFIAGGHAAMFDLPHATNLQTLAQQIYASNGVLAAVCHGPVMLPFVDDTKSPEGRSVVYGKKVTAFNSTGELVMGVSSALRERNMQDLNSLFREAGAEFVDPPTPMSDFTQVDGRIVTGVNPMSAKSTAEAAIKVSQSLRKT*

>SpGLYIII3

MSIAKGKNALLVASSYYGPFYPDGKNTGVHFSELLIPYNVFKKAGFNVQFVSENGSYKFDDHSIEESKLGDFERKVFNDKNDDFWTNLNNMKKASDIVGKDYQLLFVAGGHAAMFDLPKATNLQAVAREVFTNGGVLSAVCHGPVLLANVKNPQSVEGKTVVYHKHVTAFNKAGEEKMGVMDELKKRGMKSLNEIFAEAGATFIDPPNPNVNFTQIDGKIVTGVNPQSAKSTAEAAVSAL*

>SpGLYIII4

MPAKTRNVLIACSDYYGPFYKDGENTGAFFLELLHPYLVFRDACFNVDIVTESGKIQFDDHSVAGPAIDKGSKGEEFLSYDDHIASGPELSKAEKYVLENKDDMFWRIVQNSKTADEVNPDKYDIFFVAGGHATLFDFPKATNLQKLGTSIYENGGVVAAVCHGPTLLPFMKRQTSDGSVSIVCGKDVTAFDRVAEDKSKLMEALKKYNLEVLDDMLNDAGANFIKSPNPFGDFVIADGRLVTGSNPASATSTAKTALRVL*

>SpGLYIII5

MVLFMKTVQRPEHISLKSCIPFKSLQRQGIVFRLSVRMVMLADDHSISDSALSDSDKNAFKDKNNDFWKAIKNAKNASDINFSDYSIFFAAGGHGTLFDFPSATNLHKGAAKIYSMGGVIAAVCHGPVILPCIKDSTGFSIVKGKTVTAFNEIAEQQMNLMPTFEKYHFKTLNKLFQEAGSNFVDPQEPFDDFVKTDGKLVTGANPASAASTAKAALNSLNS*

>SpGLYIII6

MDERHEAAGETSEKPKVLFLLNSYYGPFYDDGDNTGVNVVDLYEAFKVFEENGFDIVIASDTGDYGFDDKSFRDPAIVDETQSIFSNPDCSLMKKLKNIARLDRLNPSDYVIVYIPGGYGCSFDFPHAKVVQDFLYRFYETKGIICAVAQANIALAYTTNSDGQALCTNRRVTGCTWKDEVQNGVLNVMNRLNFYSFGHIAENIGAIFESPPVYVEDPFIVEDGQLFTGSNTNSAKGVAMEAVRAVLNYDG*
