# Supplementary material for: Tracing the Evolution of Plant Glyoxalase III Enzymes for Structural and Functional Divergence
Source: Antioxidants (Basel). 2021 Apr 23;10(5):648. doi: 10.3390/antiox10050648 (PMC8170915; doi:10.3390/antiox10050648)
Supplement: Supplementary file 1 [file antioxidants-10-00648-s001.zip › Kumar et al Supplementary files/Kumar et al Supplementary Figure S2.pdf]

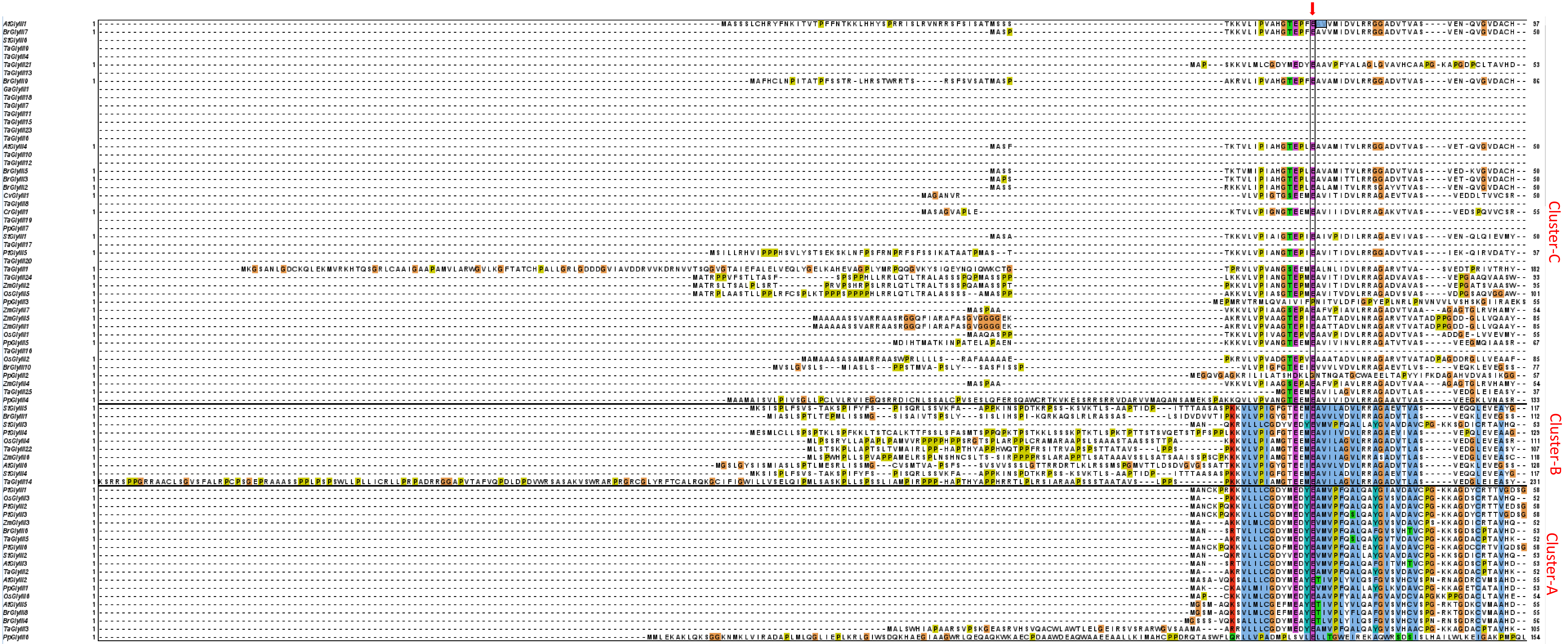
Cluster-C
Cluster-B
Cluster-A

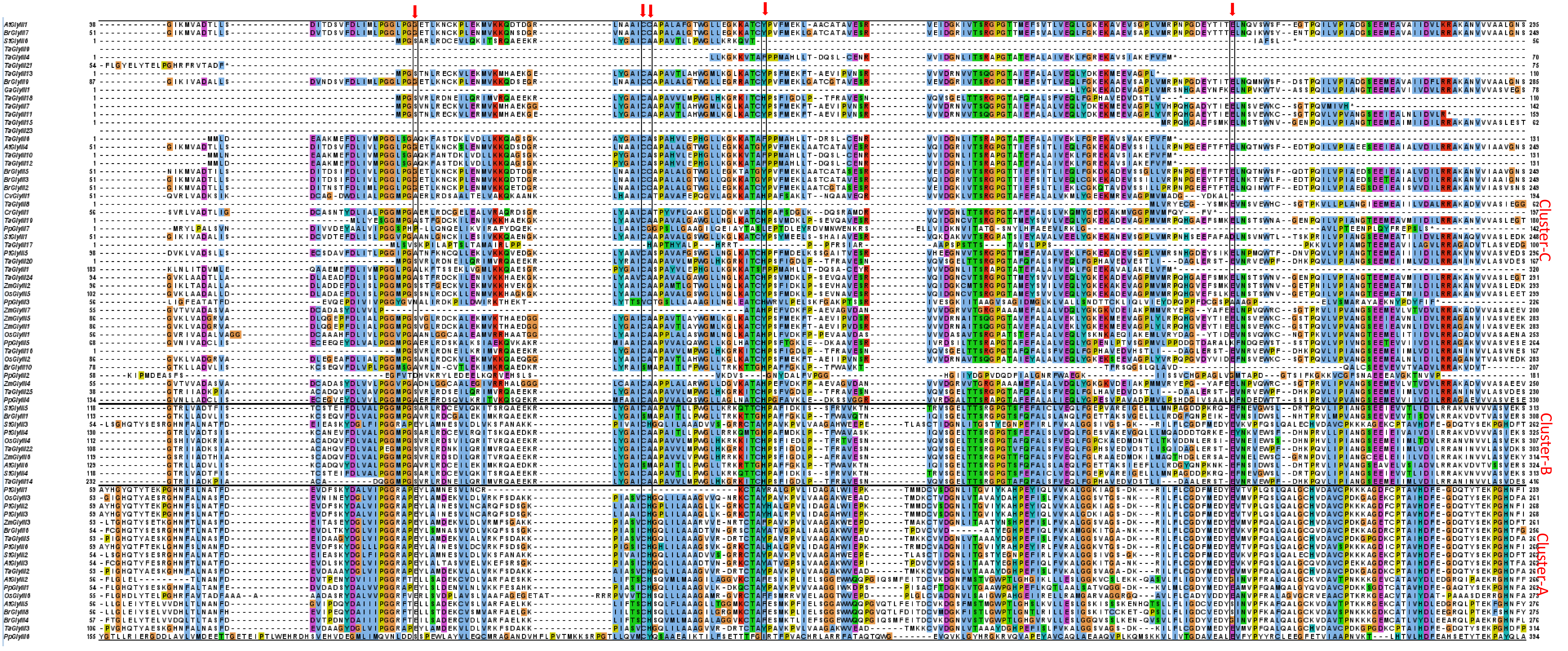
Cluster-C
Cluster-B
Cluster-A

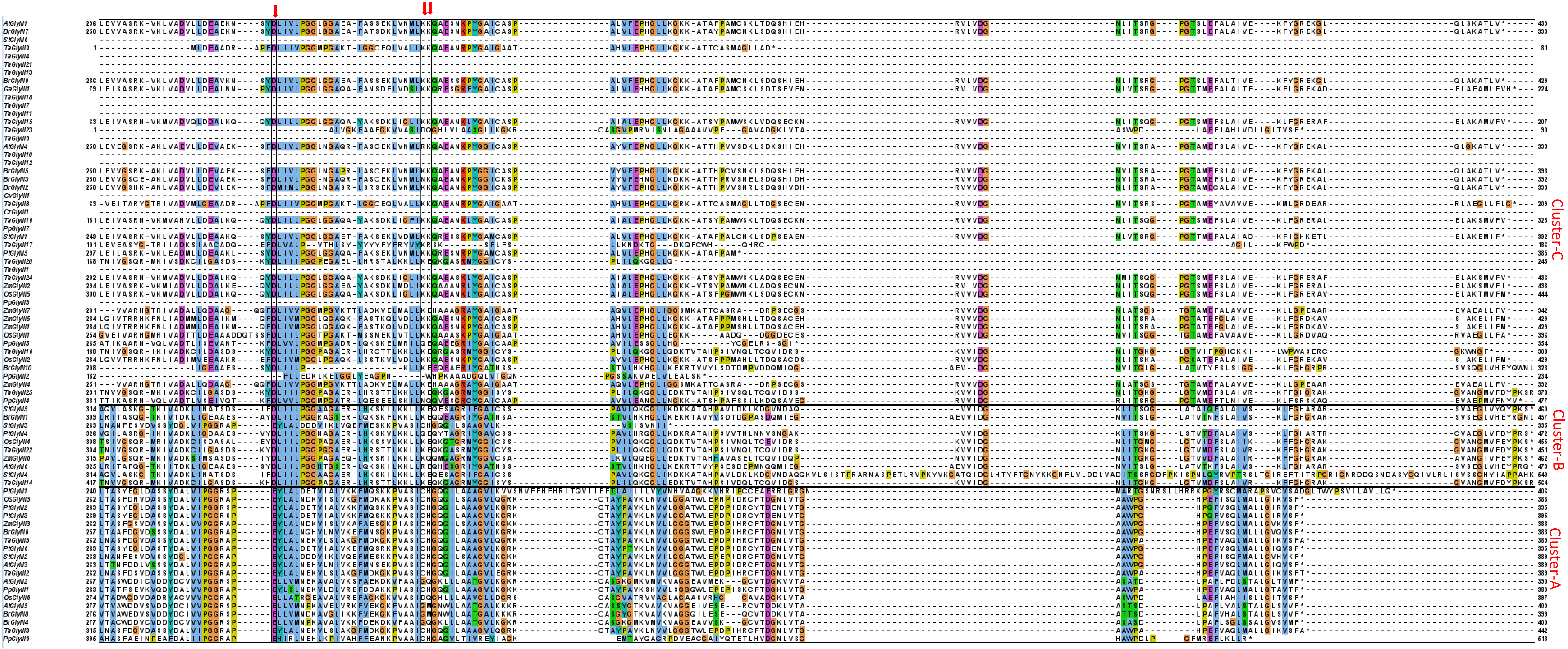
Cluster-C
Cluster-B
Cluster-A

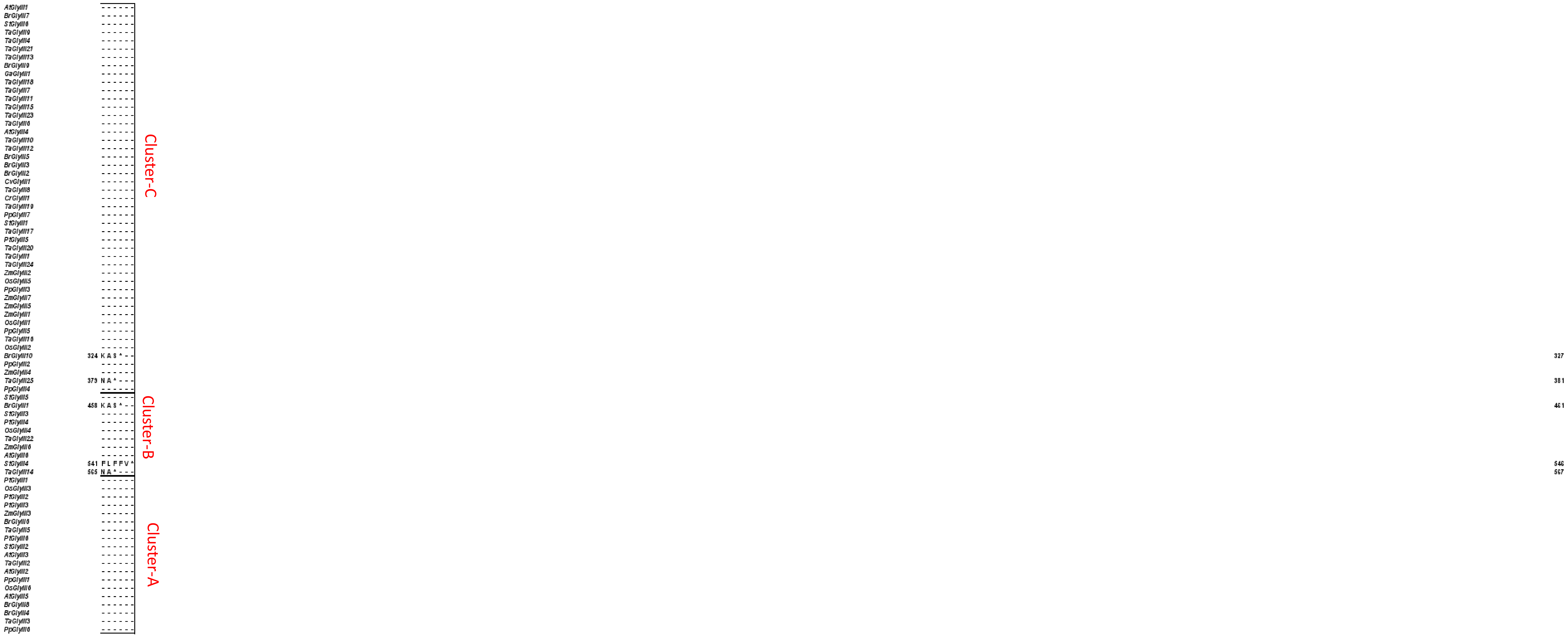
Cluster-C
Cluster-B
Cluster-A
